# PRDM16 regulates a temporal transcriptional program to promote progression of cortical neural progenitors

**DOI:** 10.1242/dev.194670

**Published:** 2021-03-17

**Authors:** Li He, Jennifer Jones, Weiguo He, Bryan C. Bjork, Jiayu Wen, Qi Dai

**Affiliations:** 1 Department of Molecular Bioscience, the Wenner-Gren Institute, Stockholm University, 10691 Stockholm, Sweden; 2 Department of Biochemistry and Molecular Genetics, College of Graduate Studies, Midwestern University, Downers Grove, IL 60515, USA; 3 Department of Genome Sciences, The John Curtin School of Medical Research, The Australian National University, 2601 Canberra, Australia

**Keywords:** PRDM16, Radial glia, Temporal identity, Neocortex, Mouse

## Abstract

Radial glia (RG) in the neocortex sequentially generate distinct subtypes of projection neurons, accounting for the diversity and complex assembly of cortical neural circuits. Mechanisms that drive the rapid and precise temporal progression of RG are beginning to be elucidated. Here, we reveal that the RG-specific transcriptional regulator PRDM16 promotes the transition of early to late phase of neurogenesis in the mouse neocortex. Loss of *Prdm16* delays the timely progression of RG, leading to defective cortical laminar organization. Our genomic analyses demonstrate that PRDM16 regulates a subset of genes that are dynamically expressed between early and late neurogenesis. We show that PRDM16 suppresses target gene expression through limiting chromatin accessibility of permissive enhancers. We further confirm that crucial target genes regulated by PRDM16 are neuronal specification genes, cell cycle regulators and molecules required for neuronal migration. These findings provide evidence to support the finding that neural progenitors temporally shift the gene expression program to achieve neural cell diversity.

## Abstract (precis)

**Summary:** During neocortical development, PRDM16 promotes temporal progression of radial glia by regulating a dynamic gene expression program. This activity ensures the transition from mid- to late-neurogenesis.

## INTRODUCTION

Radial glia (RG) in the developing mammalian cerebral cortex are neural stem cells that give rise to all excitatory neurons (Anthony et al., 2004; Kriegstein and Alvarez-Buylla, 2009). During the peak phase of neurogenesis, RG divide asymmetrically to produce a self-renewing RG and a neuron or a transit-amplifying intermediate progenitor (IP) that divides again to produce more neurons (Noctor et al., 2004). On each embryonic day (E), starting at E11.5, RG generate a new laminar layer with distinct neuronal subtypes. Layer (L) 6 neurons are born first at E12.5, followed by L5 (E13.5), L4 (E14.5) and L2/3 (E15.5) neurons. The newborn neurons migrate along the radial fiber of the mother RG, passing through and positioning on top of earlier-born neurons (Kwan et al., 2012; Angevine and Sidman, 1961; Okano and Temple, 2009). Thus, the identity and laminar position of neuronal subtypes are tightly linked to their birthdates.

The competence of progenitors becomes progressively restricted (Desai and McConnell, 2000; Gaspard et al., 2008; Gao et al., 2014; Frantz and McConnell, 1996), and the temporal identity of neural progenitors is controlled by both extrinsic and intrinsic mechanisms (McConnell and Kaznowski, 1991; Ge et al., 2006; Dennis et al., 2017; Fukumitsu et al., 2006; Shen et al., 2006; Hsu et al., 2015; Chenn and Walsh, 2002; Yoon et al., 2017; Vitali et al., 2018). Recent single cell transcriptome analyses revealed that RG at different neurogenesis stages may possess temporal gene expression programs (Telley et al., 2019; Yuzwa et al., 2017). However, the mechanisms of how such temporal expression is established remain to be explored.

In differentiating neurons, cell-specific transcription factors and their regulated transcriptional cascades further guide neuronal specification, migration and circuit assembly (Greig et al., 2013). For example, complex interplay between TBR1, FEZF2 and SATB2 guide specification of corticothalamic, subcerebral and callosal neurons in deep-, mid- and upper-cortical layers (McKenna et al., 2015; Srinivasan et al., 2012). Two related POU domain transcription factors, POU3F2 (BRN2) and POU3F3 (BRN1), are required for determining the identity and migration of upper layer neurons (McEvilly et al., 2002; Sugitani et al., 2002). Although key neuronal specification factors are used as subtype-specific markers (Molyneaux et al., 2007), the mRNAs of these factors already appear in neural progenitors (Yoon et al., 2017; Zahr et al., 2018). Whether transcription of neuronal specification genes in the RG is important for rapid daughter cell differentiation after asymmetric division remains to be demonstrated.

The choroid plexus (ChP) protects the brain via the blood-cerebrospinal fluid (CSF) barrier and regulates CSF composition via specific transfer and secretion (Johansson, 2014; Lehtinen et al., 2013). Signaling molecules in CSF (e.g. SHH, IGF1, WNT4, TGM2 and FGF2) are delivered to NSCs and influence NSC behavior (Johansson, 2014; Imayoshi et al., 2008; Lehtinen et al., 2011; Johansson et al., 2013). Mechanisms and factors controlling development of the ChP are not fully understood.

The PR domain-containing (PRDM) family protein PRDM16 is a key transcriptional regulator in diverse cell types (Aguilo et al., 2011; Chuikov et al., 2010; Kajimura et al., 2008). In brain development and homeostasis, PRDM16 was shown to control neural stem cell maintenance and proliferation (Chuikov et al., 2010; Shimada et al., 2017; Chui et al., 2020), IP proliferation (Baizabal et al., 2018), neuronal migration (Inoue et al., 2017; Baizabal et al., 2018; Chui et al., 2020) and ependymal cell differentiation (Shimada et al., 2017). PRDM16 regulates genes involved in reactive oxygen species (ROS) levels (Chuikov et al., 2010; Inoue et al., 2017; Chui et al., 2020) and epigenetic states of its bound enhancers (Baizabal et al., 2018). The PRDM16 protein (Fig. S1A) contains a PR domain that possesses intrinsic Histone H3K4 (Zhou et al., 2016) and H3K9 methyltransferase activity (Pinheiro et al., 2012), two potential DNA binding zinc-finger clusters (Nishikata et al., 2003) and interaction motifs for the co-repressors CtBP1/2. The transcriptional activity of PRDM16 is context-dependent (Chi and Cohen, 2016), as it activates gene expression when associated with activators and represses gene expression when interacting with co-repressors.

In this study, we investigated the mechanisms by which PRDM16 regulates RG property in the developing mouse brain. We show that *Prdm16* deficiency compromises the development of ChP and cortical layer lamination and that specific depletion of *Prdm16* from the forebrain resulted in similar defects in the neocortex. Although the number of RG in knockout animals appeared to be normal, there was a delay in the progression of RG from producing earlier-born L5 neurons to later-born L2-4 neurons. Our genomic data demonstrated that PRDM16 primarily represses target gene expression through modulating permissive enhancers. By integrating public scRNA-seq data with our RNA-seq and ChIP-seq analysis, we identified a subset of PRDM16-regulated genes that are dynamically expressed from early to late neurogenesis. We propose a model in which PRDM16 helps to set up the temporal transcriptional landscape of RG. As a proof of principle, we confirmed that two crucial target genes of PRDM16, *Cdkn1c* and *Flrt3*, the expression of which declines in late neurogenesis, are responsible for the regulation of progenitor proliferation and neuronal migration, respectively.

## RESULTS

### PRDM16 is required for neocortical development and ChP formation

To assess the function of *Prdm16* in the developing brain, we used three multifunctional conditional gene trap (cGT) alleles (Strassman et al., 2017) (Fig. S1B). The *Prdm16^cGT^* (*cGT*) and *Prdm16^cGTreinv^* (*cGTreinv*) mouse strains produce a null allele and will be referred as *Prdm16* KO mutants. To examine PRDM16 activity in the neocortex, we depleted *Prdm16* expression in the forebrain using the *Emx1^tm1(cre)Krj/J^* (*Emx1^IREScre^*) deleter strain (Gorski et al., 2002) and the conditional *Prdm16^cGTinv^* (*cGTinv*) strain. *cGTinv* will be referred to as *Prdm16* cKO throughout this manuscript. The *Prdm16* transcript is detectable in E9.5 brain (Fig. S1C; (Horn et al., 2011). At E13.5, PRDM16 has specific expression in the ChP and in the ventricular zone (VZ), where it colocalizes with the RG marker SOX2 (Fig. S1D). In KO animals, PRDM16 staining is lost in the entire brain (Fig. S1D), whereas in cKO mutants it is depleted in the dorsal telencephalon but remains expressed in the ventral telencephalon and the ChP (Fig. S1E).

**Fig. 1.**
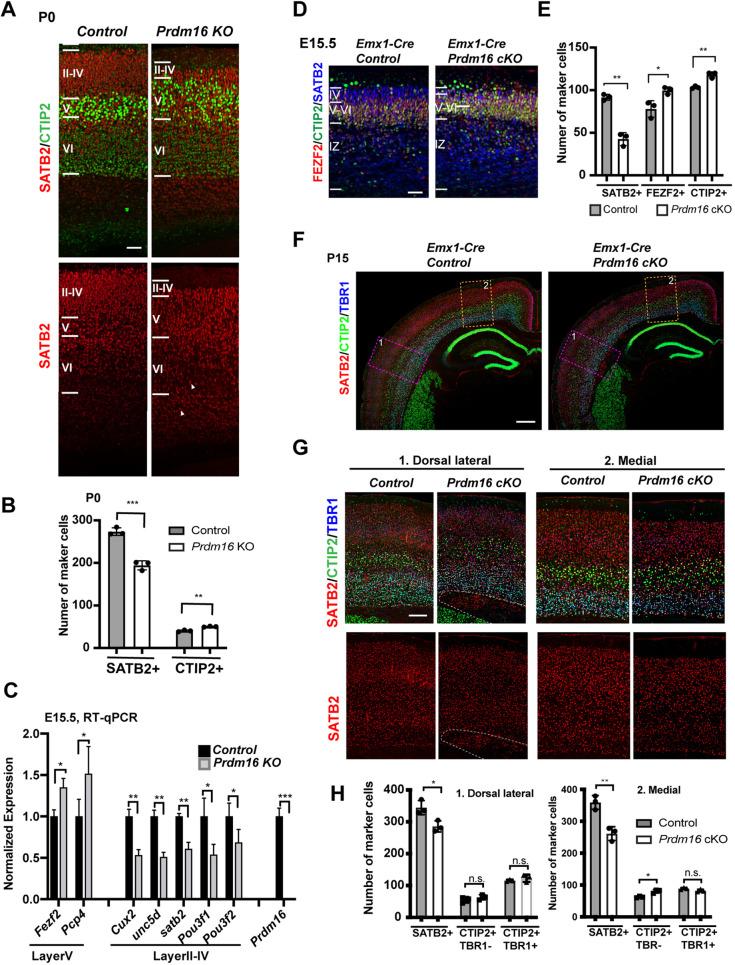
**PRDM16 regulates cortical laminar organization.** (A) Immunostaining images of P0 control and *Prdm16* KO cortices stained with the upper-layer marker SATB2 in red (top and bottom) and the mid-layer marker CTIP2 in green (top). Cortical layers are highlighted according to expression of SATB2 and CTIP2. White arrowheads point to some retained SATB2+ cells in the deep layers. (B) Quantification of the marker-positive cells in an 80 µm-wide column across the cortex (*n*=3). (C) Measurement of layer marker gene expression and *Prdm16* by RT-qPCR from E15.5 control and *Prdm16* KO cortices. (D) Images of E15.5 control and Emx1-Cre:: *Prdm16* cKO cortices show reduction of the SATB2+ layer (blue) and expansion of the CTIP2+ (green) and FEZF2+ (red) layer. (E) Quantification of the marker-positive cells in a 100 µm width column across the cortex (*n*=3). (F) Images of P15 control and *Prdm16* cKO cortices stained with SATB2 (red), CTIP2 (green) and TBR1 (blue). Dorsal lateral and medial areas are highlighted with pink and orange rectangles, respectively. (G) Higher magnification images of rectangles in F show reciprocal effects on CTIP2+ and SATB2+ layers in cKO cortices. Heterotopia tissue is highlighted by the white dashed line. (H) Quantification of the numbers of the three cell types in a 300 µm-wide column in each area (*n*=3). All data are shown as mean±s.d.; *P<0.05; **P<0.01; ***P<0.001 (two-tailed unpaired Student's *t*-test). n.s., not significant. Scale bars: 50 µm (A,D,G); 100 µm (F).

We first analyzed the cortical laminar organization of the KO brains by labeling cortical neurons with SATB2 for the upper-layer (L2-4, II-IV) and CTIP2 (also known as BCL11B) for the mid-layer (L5, V; strong CTIP2) and the deep-layer (L6, VI; weak CTIP2). At postnatal day (P) 0, mutant cortices showed expansion of the CTIP2+ layer, accompanied by thinning of the SATB2+ upper layer (Fig. 1A,B), compared with control cortices. Some SATB2+ neurons scattered inside the deep layer, indicative of failed migration. Similarly, at E15.5, when upper-layer neurons were born, the number of SATB2+ neurons decreased and the mid-layer neurons labeled with FEZF2 and CTIP2 expanded in the mutant (Fig. S1F,G). We confirmed the change using reverse transcription followed by quantitative PCR (RT-qPCR). The levels of the two L5 genes increased to ∼150%, whereas those of the L2-4 genes decreased to 50-70% (Fig. 1C), indicating that gain of mid-layer neurons roughly compensates for loss of upper-layer neurons at E15.5. Hence, the *Prdm16* KO cortex displays two types of defects: overproduction of mid-layer neurons, and reduced production and defective migration of upper layer neurons.

In *Prdm16* KO brains, the prospective ChP in the lateral and the third ventricles are dramatically reduced (Fig. S1D,H,I; Bjork et al., 2010b; Strassman et al., 2017), pointing to an essential role of PRDM16 in ChP development. Together, the phenotypic analyses in *Prdm16* KO mutant suggest that PRDM16 controls brain development in at least two brain areas, the neocortex and the ChP.

### Expression of Prdm16 in the forebrain regulates cortical laminar organization

To test a direct role of PRDM16 in cortical development, we analyzed *Emx1^IREScre^*-mediated *Prdm16* cKO mutants, in which *Prdm16* is depleted in the forebrain (Fig. S1E). The *Prdm16* cKO animals survive to adulthood, allowing examination of postnatal stages. At E15.5, *Prdm16* cKO cortices displayed defects similar to *Prdm16* KO embryos: an increase in the number of CTIP2+ and FEZF2+ neurons and a reduction in the number of SATB2+ neurons (Fig. 1D,E). At P15, the cKO cortex showed similar changes (Fig. 1F-H), and the TBR1-labeled deep-layer is unaffected. A subset of SATB2+ neurons failed to migrate and remained below the cortex, as a chunk of grey matter cells (heterotopia) (Fig. 1F,G). Thus, the forebrain depletion of *Prdm16* led to the same effects on cortical laminar organization as the null KO did: reciprocal changes of L5 and L2-4 neurons and failure of upper-layer neuron migration. This result confirms that the laminar organization phenotypes in the mutant cortex are due to loss of *Prdm16* in the forebrain.

### PRDM16 regulates the transition of mid-to-late neurogenesis

We sought to understand the causes of *Prdm16* mutant phenotypes. Given that PRDM16 is an RG-specific factor, PRDM16 may control neurogenesis through modulating intrinsic properties of RG. We reasoned that two possibilities could lead to the increase of L5 neurons and decrease of L2-4 neurons. First, if loss of *Prdm16* delayed the transition of neurogenesis from E13.5 to E14.5, mutant RG would produce L5 neurons even after E13.5, which could result in fewer L2-4 neurons. Second, if loss of *Prdm16* increased proliferation of RG at E13.5 and reduced it at E14.5 and later, more mid-layer neurons could be produced at E13.5 and fewer upper-layer neurons produced at a later time. We attempted to test these possibilities.

**Fig. 2.**
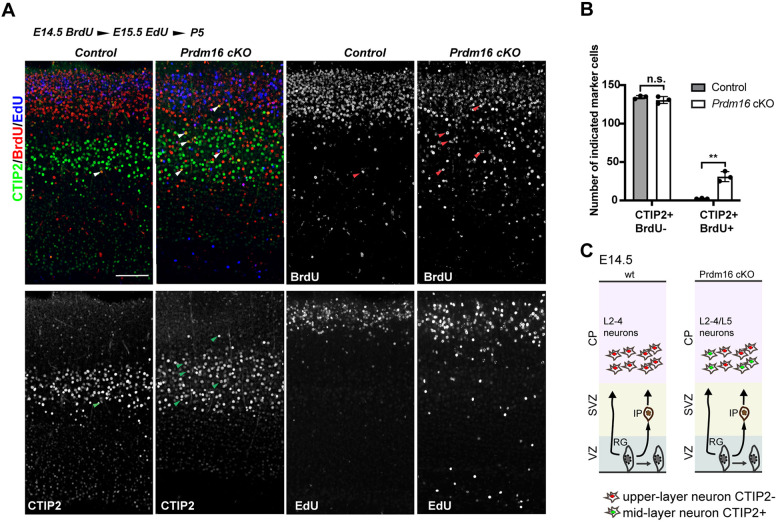
**Forebrain-specific depletion of PRDM16 delayed mid-to-late neurogenesis transition.** (A) Images of P5 control and *Prdm16* cKO cortices, stained with CTIP2, BrdU and EdU. White, green and red arrowheads point to BrdU+CTIP2+ cells. (B) Quantification of the numbers of the CTIP2+BrdU− and CTIP2+BrdU+ cells in a 300 µm-wide column across the cortex (*n*=3). All data are shown as mean±s.d.; *P<0.05; **P<0.01; ***P<0.001 (two-tailed unpaired Student's *t*-test). n.s., not significant. (C) Illustration of the progression delay: mutant RG produce CTIP2+ neurons at E14.5. Scale bar: 100 µm.

To test whether PRDM16 controls the timing of RG transition, we traced RG daughter cell fate by injecting pregnant mice with 5-bromo-2'-deoxyuridine (BrdU) at E14.5 and 5-ethynyl-2′-deoxyuridine (EdU) at E15.5, and examined the distribution and cell identity of BrdU and EdU cells in the P5 cortex. CTIP2+ L5 neurons are born at E13.5 and should not be labeled with BrdU or EdU (Desai and McConnell, 2000; Gaspard et al., 2008). This was seen in the control cortex (Fig. 2A,B). In contrast, supernumerary CTIP2+BrdU+ neurons appeared in the mutant cortex, suggesting CTIP2+ neurons were produced at or after E14.5 in the mutant. Notably, the number of the CTIP2+BrdU− cells did not change in the mutant, indicating that production of CTIP2+ neurons before E14.5 was normal. These results demonstrate that some of *Prdm16* mutant RG failed to transit from E13.5 to E14.5 and continued to produce CTIP2+ neurons at E14.5 (Fig. 2C).

BrdU+ and EdU+ cells were found in the heterotopia and the deep layer (Fig. S2A-C), confirming failed migration of some of the upper-layer neurons. None of the CTIP2+BrdU+ cells were retained in the deep layer (Fig. 2A) or the heterotopia (Fig. S2B), suggesting that even the latter-produced CTIP2+ neurons migrate normally and that the migration failure is specific to upper-layer neurons.

### PRDM16 promotes indirect neurogenesis and production of IPs

**Fig. 3.**
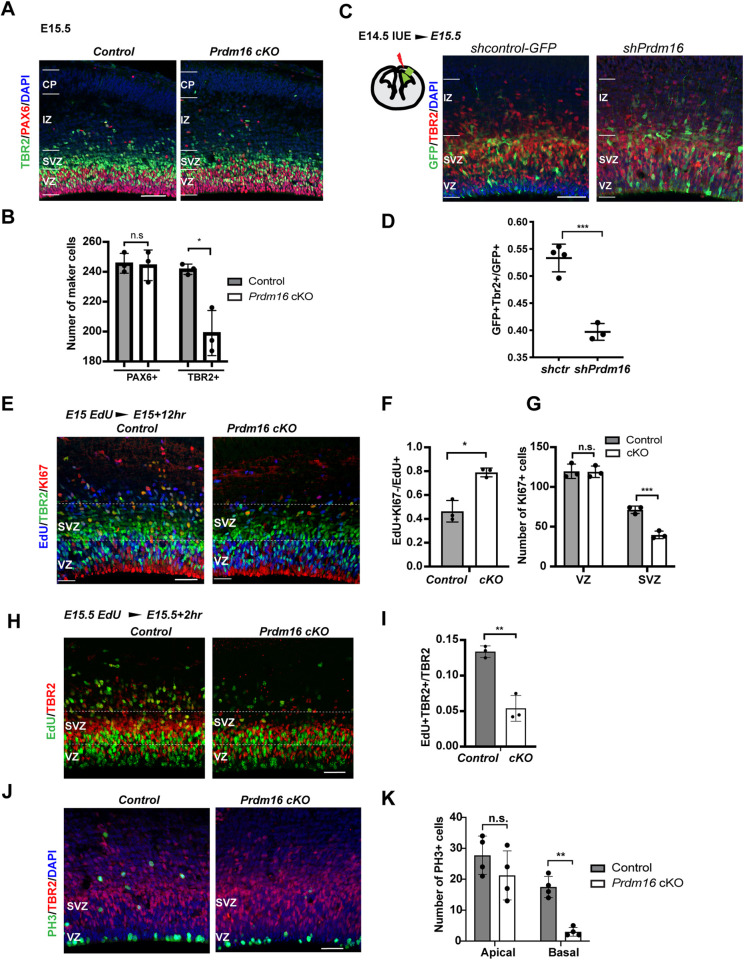
**PRDM16 promotes IP cell production during late cortical neurogenesis.** (A) Images of E15.5 control and *Prdm16* cKO cortices, stained with the IP marker TBR2 (green), the RG marker PAX6 (red) and DAPI (blue). (B) Quantification of the marker-positive cells in a 200 µm-wide column across the cortex (*n*=3). (C) Images of E15.5 cortices injected with the shcontrol-GFP vector or *shPrdm16*-GFP by *in utero* electroporation, showing GFP (green), TBR2 (red) and DAPI (blue). (D) Quantification of the fraction of TBR2+GFP+ cells among GFP+ cells. Control: *n*=4; *shPrdm16*: *n*=3. (E) Images of E15.5 control and *Prdm16* cKO cortices, stained with EdU (blue), TBR2 (green) and KI67 (red). White dashed lines highlight the SVZ. (F) Quantification of the fraction of EdU+Ki67− over EdU+ cells (*n*=3) within a 250 µm-wide column. (G) Quantification of KI67+ cells within a 250 µm-wide column (*n*=3). (H) Images of E15.5 control and *Prdm16* cKO cortices following a 2 h EdU pulse labeling, stained with EdU (green) and TBR2 (red) antibodies. (I) Quantification of the fraction of EdU+TBR2+ over TBR2+ cells within a 250 µm-wide column. (J) Images of E15.5 control and *Prdm16* cKO cortices, stained with PH3 (green), TBR2 (red) and DAPI (blue). (K) Quantification of the fraction of PH3+ at the apical surface and the basal side of the ventricle within a 250 µm-wide column. All data are shown as mean±s.d.; *P<0.05; **P<0.01; ***P<0.001 (two-tailed unpaired Student's *t*-test). n.s., not significant. Scale bars: 50 µm.

Next, we checked the progenitor populations in *Prdm16* mutant. We examined RG and IP cell counts at E15.5 by labeling RG with PAX6 and IPs with TBR2 (EOMES). There was a significant reduction in the number of IPs in the cKO cortex, whereas PAX6+ RG were not affected (Fig. 3A,B), suggesting that PRDM16 is required for IP cell production.

RG divide and produce neurons directly (direct neurogenesis) or indirectly through IP cells (indirect neurogenesis). Although both division modes are present throughout cortical neurogenesis, indirect neurogenesis becomes more predominant in later stages (Yuzwa et al., 2017) (Vitali et al., 2018). As the IP cell number is reduced in the *Prdm16* mutant, we examined whether PRDM16 promotes indirect neurogenesis. We electroporated a short-hairpin RNA (shRNA) construct targeting *Prdm16* (Bjork et al., 2010a) and an empty shRNA construct to wild-type E15.5 brains *in utero*. *shPrdm16*-transfected cells led to significantly fewer TBR2+ cells compared with those transfected with the shRNA vector (Fig. 3C,D). These results suggest that PRDM16 promotes indirect neurogenesis.

The duration of S phase to M phase in E15 RG is 6 h (Takahashi et al., 1995). If a daughter cell of RG has exited the cell cycle, this cell will be labeled with EdU but not with the cell cycle marker KI67 (MKI67) after 6 h. EdU+KI67− cells will reflect the collection of post-mitotic neurons generated from direct neurogenesis and cells that do not re-enter the cell cycle. We injected EdU into pregnant mice with embryos at E15 and analyzed the brains after 12 h. Compared with the control, the *Prdm16* cKO cortex displayed a significant increase in the percentage of EdU+KI67− cells among EdU+ cells in the VZ/subventricular zone (SVZ) region, indicating more cells exiting the cell cycle in the mutant (Fig. 3E,F).

Moreover, we detected a significant decrease of KI67+ cells specifically in the mutant SVZ (Fig. 3G), suggesting that PRDM16 also regulates IP cell proliferation. To confirm this, we injected animals with EdU at E15.5 and collected brains 2 h after. The fraction of EdU+TBR2+ cells over TBR2+ cells is significantly less in the mutant compared with the control (Fig. 3H,I; Fig. S3), showing fewer TBR2+ cells in S phase in the mutant. In addition, mitotic cells labeled with phosphorylated Histone 3 (PH3) were reduced in the basal domain of the ventricle in mutant cortices (Fig. 3J,K). This evidence indicate that loss of *Prdm16* affects IP cell proliferation.

We next examined cell counts and proliferation of RG and IP cells at E13.5. In contrast to E15.5, neither the PAX6+ nor the TBR2+ cells showed a significant change in cKO cortices (Fig. S4A,B). Staining with PH3 confirmed no change in the number of mitotic cells (Fig. S4C,D). EdU labeling from E13 to E13.5 showed that the fraction of EdU+KI67− cells over all EdU+ cells in cKO cortices appeared to be similar to the control (Fig. S4E,F), indicating that cells exit the cell cycle normally in the mutant at this stage. Another RG marker, SOX2, did not show any change (Fig. S4G). Together, these results suggest that PRDM16 regulates RG neurogenesis in a stage-specific manner: first, it promotes the temporal transition of RG between E13.5 and E14.5; second, it promotes indirect neurogenesis and IP proliferation during late-neurogenesis.

We also attempted to check the effects by overexpressing PRDM16. Surprisingly, overexpressing PRDM16 resulted in severe migration defects and fewer TBR2+ IP cells, compared with the control injection (Fig. S4H-J). This means that the level of PRDM16 is important for regulating neuronal migration and IP cell production.

### PRDM16 modulates levels of neuronal specification genes in RG

**Fig. 4.**
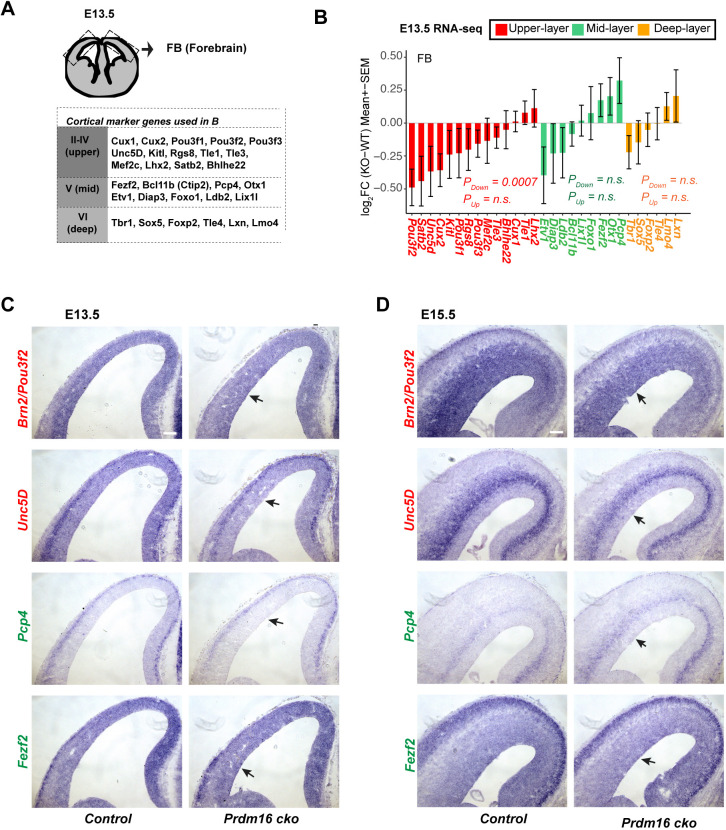
**Depletion of PRDM16 led to defective gene expression in the developing cortex.** (A) Schematic of the RNA-seq tissue and a list of layer marker genes described in the study. (B) Log2 fold-change in KO versus wild-type (WT) of upper-, mid- and deep-layer genes. A gene set test shows the upper-layer markers are significantly downregulated. (C,D) *In situ* hybridization for Pou3f2, Unc5D, Pcp4 and Fezf2 in control and *Prdm16* cKO mutant cortices at E13.5 (C) and E15.5 (D). Black arrows indicate ventricle surface. Scale bars: 100 µm.

PRDM16 is a transcription factor specifically expressed in the RG in the neocortex. Because the cell fate change in *Prdm16* mutants occurs at the transition between mid-layer neuron production and upper-layer neuron production (Fig. 2), a direct regulatory role of PRDM16 on RG gene expression may be crucial at E13.5. To test this, we generated RNA-seq data from E13.5 control and KO mutant forebrains (FB) (Fig. 4A), and identified 35 downregulated and 47 upregulated genes in KO versus control (cutoff: *P*-value<0.05 and fold-change>1.5-fold; Fig. S5A). A recent study published the RNA-seq data of sorted RG, IPs and cortical neurons (CNs) from E15.5 control and *Emx1^IREScre^-*mediated *Prdm16* cKO cortices (Baizabal et al., 2018). We confirmed that most of the deregulated genes in the mutant FB were deregulated in the E15.5 mutant RG (Fig. S5A,B). Consistent with RG-specific expression of *Prdm16*, more genes changed expression level in mutant RG than those in IPs and neurons.

As the *Prdm16* mutant cortex displays abnormal laminar layer organization, we next examined expression of layer marker genes (Molyneaux et al., 2007; Zahr et al., 2018) in the RNA-seq data (Fig. 4A). Several upper-layer genes showed decreased expression in the E13.5 KO FB (Fig. 4B). Using a Limma-based gene set testing (see Materials and Methods), we confirmed a significant downregulation of the upper-layer markers as a gene set (*P*_downregulation_=0.0007). As the upper-layer neurons are not specified at E13.5, the expression change of upper-layer genes likely occurred in progenitor cells. In contrast, neither the mid-layer nor the deep-layer genes displayed significant change as a group in the KO FB, although the expression of three genes, *Pcp4*, *Otx1* and *Fezf2*, showed mild increase (Fig. 4B). We also analyzed changes of the layer-specification genes in the published E15.5 RNA-seq data (Baizabal et al., 2018) and observed a similar trend: the upper-layer genes are significantly downregulated (Fig. S5B) and the mid-layer genes as a group showed significant upregulation in mutant RG and IPs, but not in CN, consistent with our hypothesis that these genes are regulated by PRDM16 in progenitor cells.

At E13.5 the mid- and deep-layer neurons are specified, and many of the mid-layer genes are also expressed in the deep-layer. These may mask cell-type specific expression change for the mid- and deep-layer genes from whole FB RNA-seq data. To verify cell-specific changes, we examined expression of *Pou3f2* and *Unc5d*, two upper-layer genes, and *Pcp4 and Fezf2*, two mid-layer markers in Emx1-Cre *Prdm16* cKO mutants using RNA *in situ* hybridization (Fig. 4C,D). The signal intensity of *Pou3f2* and *Unc5d* was reduced in the mutant VZ/SVZ area at both stages (Fig. S5C), confirming the reduction of their expression in mutant progenitors. Expression of the mid-layer gene *Pcp4* showed a mild but significant increase in the mutant VZ/SVZ at E15.5. *Fezf2* displayed a trend of increased expression but is shy of significance. This suggests that derepression of *Fezf2* may be limited in a small population of cells. These results demonstrate that neuronal genes are already expressed in progenitors and that the normal expression of these genes is disrupted by *Prdm16* depletion.

### PRDM16 mainly functions as a transcriptional repressor in RG

**Fig. 5.**
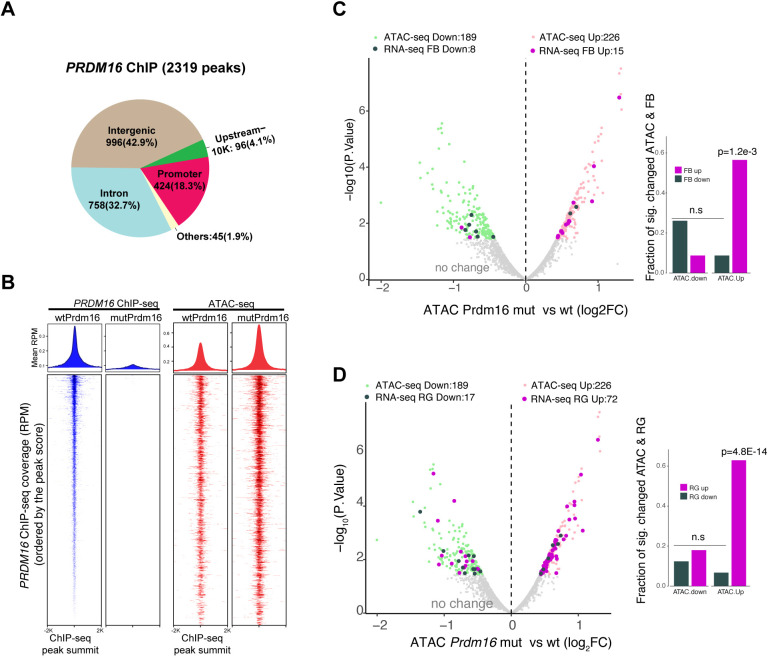
**PRDM16 represses gene expression through binding to permissive enhancers.** (A) Genomic distribution of PRDM16 ChIP-seq peaks. (B) PRDM16 ChIP-seq peaks in correlation with the ATAC-seq signal in wild-type and mutant cortices. Both types of signals are centered at ChIP-seq peak summits. Top: metaplots showing distribution of signal intensity (reads per million; RPM); bottom: heatmaps showing each ChIP-seq peak in correlation with its ATAC-seq signal. (C,D) Volcano plots showing significantly increased (light pink) or decreased (light green) ATAC-seq signal in mutant versus control at PRDM16-bound loci (FDR≤0.2). The associated genes that had expression change in the mutant FB (C) or mutant RG (D) are indicated in purple (upregulated) or dark-green (downregulated). The bar plots on the right measure the fraction of genes that changed expression level and ATAC-seq intensity at the PRDM16-bound sites, showing that the upregulated genes are associated with increased ATAC-seq signal.

To determine direct targets of PRDM16 in the developing brain and investigate how the targets are regulated, we performed chromatin immunoprecipitation followed by deep sequencing (ChIP-seq) from E13.5 heads of wild-type and *Prdm16* KO animals. Using an Irreproducibility Discovery Rate (IDR) pipeline (see Materials and Methods), we identified 2319 confident peaks (IDR<5%), of which 42.9% were mapped to intergenic regions, 32.7% to introns and only 18.3% close to promoters. This indicates that PRDM16 mainly targets distal enhancers (Fig. 5A). Gene Ontology (GO) analysis shows that PRDM16-bound genes are enriched for nervous system development, migration signaling and RG function (Fig. S6A,B). We also compared our E13.5 whole-head ChIP with the published E15.5 cortex ChIP data (Baizabal et al., 2018), and found that ∼30% (760) of the E13.5 peaks overlap with the E15.5 peaks (Fig. S6C). The overlapping sites represent continuous binding by PRDM16 between E13.5 and E15.5.

Transcriptional activity of PRDM16 is context-dependent (Chi and Cohen, 2016). We asked how the targets in the neocortex are regulated by PRDM16. To address this, we examined chromatin accessibility of PRDM16-targeted enhancers from E13.5 control and mutant cortices by using Assay for Transposase-Accessible Chromatin using sequencing (ATAC-seq) (Buenrostro et al., 2013). High ATAC-seq signal in the genome correlates with active cis-regulatory elements (Daugherty et al., 2017). To examine accessibility of PRDM16-bound regions, we plotted mean reads per million (RPM) of PRDM16 ChIP peaks in a metaplot and ranked the peaks in a heatmap, and then compared the ATAC-seq signal intensity in the wild type and mutant for each peak (Fig. 5B). The PRDM16-bound regions display high ATAC-seq intensity, suggesting that these regions are regulatory sites. Interestingly, the ATAC-seq signal at these sites became higher in the *Prdm16* mutant, suggesting that loss of *Prdm16* led to increased chromatin accessibility at PRDM16 binding sites. We quantified changes of ATAC-seq coverage on the PRDM16 ChIP-seq peaks between control and mutant and found that 226 and 189 peaks showed increased and reduced coverage, respectively [false discovery rate (FDR)<0.2 and fold-change>1.4-fold] (Fig. 5C,D). We assessed expression changes for the gene loci that associate with accessibility changes by combining the ATAC-seq data with our E13.5 FB RNA-seq data (Fig. 5C) or the published E15.5 RG RNA-seq data (Fig. 5D). In both cases, the majority of the significantly upregulated genes in the mutant displayed increased chromatin accessibility at PRDM16 binding sites, whereas downregulated ones do not show such a trend.

We then applied a gene set testing for all PRDM16 targets as a set, and found that the targets at both E13.5 and E15.5 have a significant trend of derepression in mutant RG (*P*<0.001) and IPs (*P*<0.001), but not in CNs (*P*=0.19) (Fig. S6D), confirming that many targets are normally repressed by PRDM16. We looked at a group of genes called RG core identity genes (Yuzwa et al., 2017) that are highly expressed in RG throughout neurogenesis. We found that 20 out of the 90 RG identity genes showed deregulation in the *Prdm16* mutant FB. Only the subset bound by PRDM16 became upregulated in the mutant FB or the mutant RG and IPs (Fig. S6E,F). Validation on one of the genes, *Veph1*, by RT-qPCR and *in situ* hybridization confirmed derepression of *Veph1* in *Prdm16* mutants (Fig. S6G-I). We therefore conclude that PRDM16 primarily acts as a repressor in RG through limiting chromatin accessibility.

### PRDM16 directly represses mid-layer genes including Fezf2

**Fig. 6.**
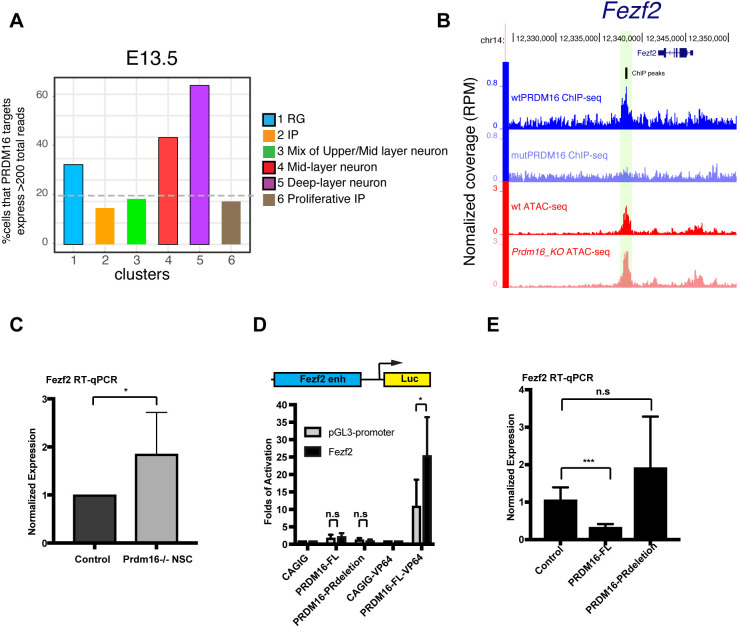
**PRDM16 represses mid-layer neuronal genes including Fezf2.** (A) Re-analysis of the cortex scRNA-seq data (Yuzwa et al., 2017) shows that PRDM16 targets are enriched in the RG, mid- and deep-layer neuron clusters at E13.5. The *y*-axis plots the percentage of cells that have summed expression of PRDM16 targets per cell >200 total normalized log2counts. (B) Screenshot of the *Fezf2* locus with a PRDM16 peak in the RG enhancer of *Fezf2*. (C) RT-qPCR from primary neural stem cell culture of control and *Prdm16* mutant cortical cells. Three pairs of control and *Prdm16* KO embryos were used. (D) Luciferase assays in N2A cells. PRDM16-FL-VP64 induced significantly higher expression of the *Fezf2* reporter than the empty pGL3-luc alone. Four biological replicates were used. (E) RT-qPCR from the N2A cells expressing pCDH-Puro (empty vector control), pCDH-PRDM16-FL or pCDH-PRDM16-PRdeletion constructs. Two independent stable lines for each construct and three technical replicates of each stable line were used. All data are shown as mean±s.d.; *P<0.05; **P<0.01; ***P<0.001 (two-tailed unpaired Student's *t*-test). n.s., not significant.

As PRDM16 represses transcription, expression of its targets in RG should be relatively low. We asked in which cell types the target genes have higher expression. To address this, we re-analyzed the published scRNA-seq data from E13.5 (Yuzwa et al., 2017) to obtain cell-type specific transcriptomes. We identified six cell-type-specific clusters and assigned cell type to each cluster based on the presence of known markers (Fig. S7A,B). Consistent with the previous finding (Zahr et al., 2018), the RG and the IP clusters express many neuronal marker genes (Fig. S7B). We then plotted the percentage of cells that contain high expression of PRDM16 targets per cell in each cluster (cutoff: total normalized reads per cell >200, see Materials and Methods; Fig. 6A; Fig. S7C). In addition to the RG cluster, the mid- and deep-layer neuron clusters showed the highest enrichment of PRDM16 targets, suggesting that many PRDM16 targets are highly expressed in mid- and deep-layer neurons.

One of the PRDM16 targets is *Fezf2* (Fig. 6B), a mid-layer neuron determinant (Molyneaux et al., 2005). PRDM16 binds to the distal enhancer of *Fezf2* known to drive *Fezf2* expression in RG (Shim et al., 2012). We confirmed that *Fezf2* is de-repressed in *Prdm16* KO mutant neural stem cells, using primary NSC culture (Fig. 6C). We then tested responsiveness of this RG enhancer to PRDM16 using a luciferase reporter (Fig. 6D). PRDM16 fused with the VP64 activation domain induced higher expression of the *Fezf2* reporter than the pGL3-promoter, confirming PRDM16 binding to this enhancer. However, PRDM16 (PRDM16-FL) or the truncated version (PRDM16-PRdeletion, lack of the PR domain) did not regulate the reporter (Fig. 6D). We reasoned that PRDM16 may require chromatin context for its regulatory activity which is lacking in transient transfection assay. To overcome this, we measured endogenous level of *Fezf2* mRNA by RT-qPCR from the N2A cell lines that express the control construct, PRDM16-FL or PRDM16-PRdeletion (Fig. 6E). PRDM16-FL could suppress *Fezf2* expression, suggesting that PRDM16 needs endogenous chromatin context or other cis-regulatory element(s) to repress *Fezf2*. Expression of *Fezf2* is not changed in cells expressing PRDM16-PRdeletion, indicating that the PR domain is essential for its repressive activity.

### PRDM16 influences temporal dynamics of gene expression in RG

**Fig. 7.**
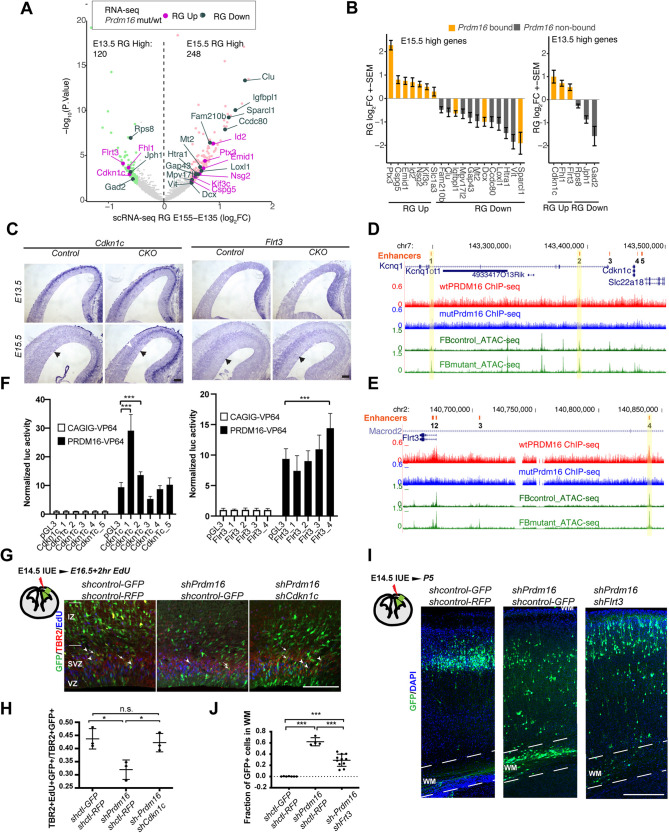
**PRDM16 regulates temporal dynamics of RG gene expression.** (A) Volcano plot showing differentially expressed genes between E13.5 and E15.5 RG (FDR<0.2 and FC>1.4-fold): 248 (light pink) higher at E15.5 and 120 (light green) higher at E13.5. The 25 significantly up- and downregulated genes in *Prdm16* cKO/wild-type (WT) RG (FDR<0.05) are highlighted in purple and dark green, respectively. (B) Expression changes of the 25 genes in *Prdm16* cKO/WT RG. The genes containing PRDM16 binding are highlighted in orange. (C) *In situ* hybridization of Cdkn1c and Flrt3 in the control and cKO brains. Black arrows indicate the VZ surface; white arrowhead points to an increased expression in IP cells. (D,E) Genome browser view of the Cdkn1c (D) and the Flrt3 (E) loci. Enhancer regions responsive to PRDM16-VP64 are highlighted. (F) Luciferase assay testing the highlighted enhancers in the Cdkn1c and the Flrt3 loci. Activity in response to PRDM16-VP64 is normalized to CAGIG and is compared between tested enhancers and the pGL3-promoter vector. The data was from three biological replicates, each of which has four technical replicates. (G,H) Knockdown tests by *shPrdm16* and *shCdkn1c* using *in utero* electroporation. G shows images from E16.5 cortices injected with the shRNA constructs and EdU, stained with EdU (blue), GFP (green) and TBR2 (red). Arrowheads highlight TBR2+GFP+EdU+ triple-positive cells; arrows highlight GFP+TBR2+ double-positive cells. H shows quantification of the fraction of GFP+EdU+TBR2+ in GFP+TBR2+ cells (*n*=3). (I,J) Knockdown tests of *shPrdm16* and *shFlrt3* using *in utero* electroporation. I shows images from P5 cortices injected with the shRNA constructs. Dashed white lines indicate white matter (WM). J shows quantification of the ratio of GFP+ cells in the white matter versus cortical plate. shControl: *n*=7; *shPrdm16*+shcontrol: *n*=4; *shPrdm16*+*shFlrt3*: *n*=11. All data are shown as mean±s.d.; *P<0.05; **P<0.01; ***P<0.001 (two-tailed unpaired Student's *t*-test). n.s., not significant. Scale bars: 100 µm.

RG produce mid-layer neurons at E13.5 and generate upper-layer neurons from E14.5 to E16.5. We hypothesized that the gene expression program of E13.5 RG differs from that of E15.5 RG to confer temporal identity. As *Prdm16* mutants show a delay of RG transition from mid- to late-neurogenesis, PRDM16 may influence the dynamics of gene expression in RG. To test this, we determined dynamically expressed genes in RG between E13.5 and E15.5, by performing differential gene expression analysis between the E13.5 and the E15.5 RG clusters from the published scRNA-seq data (see Materials and Methods). We found that 120 and 248 genes showed higher expression at E13.5 and at E15.5, respectively (FDR<0.2, FC>1.4-fold) (Fig. 7A). We then examined expression changes of these genes in *Prdm16* mutant RG [*Prdm16* cKO RNA-seq data from Baizabal et al. (2018)], and noticed that 25 of them showed significant changes (FDR<0.05) (Fig. 7B; Fig. S8A). Among these genes, the upregulated genes all contain PRDM16 ChIP peaks, consistent with the repressive role of PRDM16 on gene expression.

In the *Prdm16* mutant cortex, the transition to late neurogenesis is disrupted and many of the upper-layer neurons fail to migrate. Thus, we focused on the genes that display higher expression at E13.5 than E15.5. Among three of these genes, *Cdkn1c* encodes the cell cycle regulator p57^KIP2^ that suppresses progenitor proliferation in early neurogenesis (Mairet-Coello et al., 2012). *Cdkn1c* is more strongly expressed in IP than in RG (Fig. S8B,C), suggesting that downregulation of *Cdkn1c* in later stages may be required to allow higher proliferation of IPs. Another target gene *Flrt3* encodes fibronectin leucine rich transmembrane protein 3, a repulsive cue for the UNC5 family receptors in guiding cell migration (Yamagishi et al., 2011). Expression of *Flrt3* is also significantly upregulated in *Prdm16* mutant RG (Fig. S8D). As Unc5d is expressed in upper-layer neurons, the action between Unc5d and Flrt3 provides a possible mechanism specific for upper-layer neuron migration.

To validate the prediction from the bioinformatic analysis, we examined expression of *Cdkn1c* and *Flrt3* in the control and cKO cortices by RNA *in situ* hybridization. Both genes displayed higher expression in RG at E13.5 than E15.5 (Fig. 7C; quantified in Fig. S8E). Expression of *Cdkn1c* is undetectable in the control cortex at E15.5, but became stronger at the VZ/SVZ area in the mutant. Similarly, expression of *Flrt3* increased in the mutant VZ/SVZ compared with the control. We then screened the *Cdkn1c* and the *Flrt3* gene loci for candidate enhancers that can be directly bound by PRDM16. Using a luciferase reporter assay in N2A cells, we identified two distal enhancers for *Cdkn1c* and one enhancer for *Flrt3* that responded to ectopically expressed PRDM16-VP64 fusion protein (Fig. 7D-F). Thus, PRDM16 is able to bind regulatory regions of the predicted target genes.

To test whether *Cdkn1c* and *Flrt3* are important for PRDM16 regulation on progenitor proliferation and neuronal migration, respectively, we examined their genetic interactions with *Prdm16*. We first designed shRNA constructs to specifically knockdown *Cdkn1c* or *Flrt3*, and confirmed the knockdown efficiency of these constructs in cultured cells (Fig. S8F-G). We then electroporated *shPrdm16* alone and *shPrdm16* in combination with *shCdkn1c* or *shFlrt3* into wild-type E14.5 brains *in utero*. To examine the effects on IP proliferation, we also injected EdU 2 h before harvesting the E16.5 brains. Similar to reduced IP proliferation in *Prdm16* cKO mutants, *shPrdm16* transfection decreased the fraction of GFP+TBR2+EdU+ cells in GFP+TBR2+ cells compared with the control transfection (Fig. 7G,H; Fig. S9). Additional knockdown of *Cdkn1c* could partly restore proliferative IPs, suggesting that *Cdkn1c* is an important target gene responsible for progenitor proliferation.

**Fig. 8.**
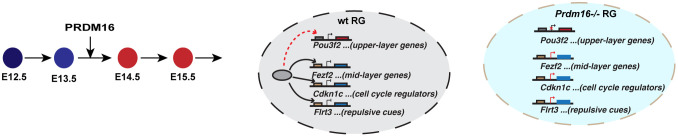
**A model summarizing PRDM16 regulation on temporal dynamics of RG.** PRDM16 regulates three classes of genes encoding mid-layer determinants, stage-specific cell-cycle regulators and migration cues. The solid black and the dashed red lines denote direct and indirect regulation by PRDM16, respectively.

To score migration defects, we harvested P5 brains and analyzed the distribution of the transfected cells labeled with GFP. As expected, *shPrdm16* treatment led to upper-layer neurons remaining in the white matter (Fig. 7I,J; Fig. S8H), whereas *shFlrt3* co-injection significantly suppressed this effect. This result confirms that PRDM16 controls upper-layer neuron migration at least partly through regulating expression of *Flrt3*. Together, our bioinformatic analyses and functional validation demonstrate that PRDM16 regulates expression of a subset of temporally-dynamic genes that mediate its role in promoting temporal transition of RG (Fig. 8).

## DISCUSSION

Our results demonstrate that PRDM16 is a crucial transcriptional regulator that controls the gene expression program of RG during cortical neurogenesis. To ensure the timed progression of RG between early and late phases of neurogenesis, PRDM16 regulates a stage-specific gene expression program that includes at least three classes of target genes (Fig. 8): (1) mid-layer determinants to allow timely upregulation of upper-layer genes in RG; (2) cell cycle inhibitors to allow higher proliferation of IPs at later neurogenesis; (3) genes encoding guidance cues needed for upper-layer neuronal migration.

Baizabal et al. recently reported that PRDM16 regulates upper-layer neuron production and migration, but found no effect on mid-layer neurons (Baizabal et al., 2018). However, we demonstrated that there is a prolonged production of mid-layer neurons in the *Prdm16* cKO cortex in addition to its effects on upper-layer neurons. The discrepancy may result from the methods used to assess cell fates. When assessing neuronal population in the cortex, we distinguished L5 mid-layer neurons from L6 deep-layer neurons, whereas Baizabal et al. assessed L5 and L6 neurons as a whole population. Our RT-qPCR and *in situ* hybridization assays provide additional evidence showing the reciprocal changes of mid-layer and upper-layer marker genes. How RG change their temporal identity is an interesting question. We found that a subset of RG-specific genes show dynamic expression between mid- to late- neurogenesis and demonstrate that timely control of gene expression by PRDM16 is important for temporal progression to late neurogenesis.

### Temporal progression of RG regulated by PRDM16

The understanding of temporal control of RG has been augmented over the years. A number of transcription factors and epigenetic regulators have been shown to control the timing of cortical neuronal specification (McConnell and Kaznowski, 1991; Ge et al., 2006; Dennis et al., 2017; Fukumitsu et al., 2006; Shen et al., 2006; Hsu et al., 2015; Chenn and Walsh, 2002). Progressive hyperpolarization of the cell membrane of RG also regulates sequential generation of neuronal subtypes through modulating Wnt signaling (Vitali et al., 2018). Post-transcriptional mechanisms, including translational repression (Zahr et al., 2018) and the N6-methyladenosine (m6A) RNA modification (Yoon et al., 2017), promote RG progression through preventing precocious production of neuronal proteins. However, many questions remain. For example, the gene expression program in RG is primed for daughter cell genes, but how is the priming status of RG established? How does the pre-established transcriptional program impact daughter cell fate? We found that PRDM16 regulates two aspects of RG temporal identity, the balance of direct and indirect neurogenesis and the transition from mid- to late-neurogenesis. *Prdm16* mutant RG display abnormal expression of mid- and upper-layer genes and several temporally-dynamic genes involved in proliferation (*Id2*, *Cdkn1c*) and migration (*Flrt3*, *Dcx*, *Sparcl1*). These results suggest that PRDM16 is involved in setting up the primed gene expression program of RG.

PRDM16 is expressed throughout cortical neurogenesis. An important question is how its activity is triggered at the onset of mid-to-late neurogenesis transition. Interestingly, *Prdm16* co-clusters with *Slc1a3*, a regulator of metabolism of glutamate and ion flux (Vandenberg and Ryan, 2013). It will be of interest to test a potential functional link between SLC1A3 and PRDM16 in integrating extrinsic and intrinsic signals. Alternatively, PRDM16 uses other mechanisms to sense environmental cues. One such candidate is the level of ROS. It has been reported that PRDM16 integrates ROS signaling with stem cell behavior (Inoue et al., 2017; Chui et al., 2020; Chuikov et al., 2010; Corrigan et al., 2018).

Many ROS-related genes were altered in the *Prdm16* mutant cortex (Chui et al., 2020). Chui et al. reported that changes on these genes influenced RG property in the *Prdm16* mutant cortex, in which proliferation of mutant RG accelerated at E13.5 but decreased at E15.5. However, in our study we did not detect similar proliferation defects in *Prdm16* mutant RG. The discrepancy could be due to the different *flox* lines used in these two studies: Chui et al. used a *flox* line that removes exon 9 of the *Prdm16* gene (Cohen et al., 2014), whereas our *Prdm16^cGTinv^* (*cGTinv*) strain stops transcription after exon 2 (Strassman et al., 2017).

Interestingly, Hamlet, the ortholog of PRDM16, controls the temporal identity of intermediate progenitors in *Drosophila* neuroblast lineage (Eroglu et al., 2014), suggesting that the role of the PRDM16 proteins is evolutionarily conserved.

### Transcriptional activities of PRDM16

PRDM16 can act as a repressor or an activator depending on its associated partners (Chi and Cohen, 2016). We showed that PRDM16-bound genes have a trend of derepression in the *Prdm16* mutant, indicating its repressive role in the neocortex. The fact that many of the PRDM16 targets are expressed in RG suggest that repression by PRDM16 is not to fully silence genes but to maintain gene expression at the right level. In support of this, PRDM16 binding associates with open chromatin. Moreover, we do not rule out the possibility of PRDM16 being an activator, as our ChIP- and RNA-seq data identified a small subset of genes that show PRDM16 binding and downregulation in the mutant.

The PR domain of PRDM16 is essential in repressing *Fezf2*. Baizabal et al. showed that PRDM16 without the PR domain failed to rescue target gene derepression (Baizabal et al., 2018). The PR domain of PRDM16 was shown to be essential in suppressing MLLr1 leukemia via intrinsic H3K4 methylation activity (Zhou et al., 2016). We did not observe global changes of H3K4me3 or H3K4me2 levels in the mutant cortex by immunostaining (Fig. S10). In agreement with this result, Baizabal et al. did not detect a significant change of H3K4 methylation levels using ChIP-seq (Baizabal et al., 2018). Future studies are needed to address the mechanistic nature of how the PR domain or any other domain contributes to the function of PRDM16 in the neocortex.

*Prdm16* is among the many genes deleted in human 1p36 microdeletion syndrome, a disorder that displays a wide variety of disease conditions. According to the function of PRDM16 in normal development, loss of *Prdm16* might contribute to several problems including the craniofacial and cardiac defects and hydrocephalus of the syndrome (Bjork et al., 2010b; Arndt et al., 2013; Shimada et al., 2017). Our findings, along with the study by Baizabel et al., defined a mechanism by which *Prdm16* loss-of-function contributes to the formation of heterotopia, a neurodevelopmental disorder that leads to severe mental retardation and seizures that were also seen in the 1p36 syndrome. More mechanistic insights of PRDM16 function in development will increase our understanding of its pathological function in diseases.

## MATERIALS AND METHODS

### Animals and processing

All animal procedures were approved by the Swedish agriculture board (Jordbruks Verket) with document number Dnr 11553-2017 and the MWU Institutional Animal Care and Use Committee. The *Prdm16*^cGT^ and *Prdm16^cGTreinv^* mice (Strassman et al., 2017) were maintained by outcrossing with the FVB/NJ line. *B6.129S2-Emx1tm1(cre)Krj/*J (*Emx1^IREScre^*) (Gorski et al., 2002) were used to generate conditional gene trap knockout animals as previously described (Strassman et al., 2017).

Plasmids used for *in utero* electroporation include shRNA sequences targeting *Flrt3* or *Cdkn1c* in the pll3.7-IresdsRed vector (Rubinson et al., 2003) and *shPrdm16*-GFP (#1) (Bjork et al., 2010a). *In utero* electroporation of DNA into C57J/B6L mice was performed as previously described (Saito and Nakatsuji, 2001; Saito, 2006). Briefly, timed pregnant mice with E14.5 embryos were deeply anesthetized using isoflurane and the uterine horns were exposed. We microinjected ∼1ul of plasmid DNA (3 μg/μl) spiked with Fast green (Sigma-Aldrich) into the lateral ventricles of each embryo using a glass micropipette (Drummond Scientific). Five 50 ms pulses of 40-45 mV with 950 ms intervals were delivered across the uterus with two 9 mm electrode paddles positioned on either side of the head (BTX, ECM830). As a standard procedure, the uterus was placed back in the abdominal cavity, the wound was surgically sutured and wound clips were placed. The whole procedure was performed on a warm heatpad. The health of the animals was monitored 24- and 48-h post-surgery.

### Molecular cloning

The pCAGIG plasmid (Addgene plasmid #11159) was inserted with a fragment encoding a nuclear localization signal (NLS) and 3×Flag in the EcoRI site. To make pCAGIG-Prdm16-FL or pCAGIG-Prdm16-PRdeletion, the full-length open reading frame (ORF) or the truncation that lacks the coding sequence for amino acids 2-180 of *Prdm16* was PCR amplified from MSCV-Prdm16 (Addgene plasmid #15504) and inserted between the EcoRI and XhoI sites in pCAGIG-NLS-Flag. The VP64 fragment was then inserted to the XhoI site of pCAGIG-Prdm16 to make pCAGIG-FL-VP64.

The plasmids used for making stable cell lines, pCDH-Prdm16 and pCDH-Prdm16-PRdeletion, were generated as follow: The Prdm16 FL ORF, the PR-deletion coding sequences or the NLS-3×Flag was digested from their pCAGIG plasmids and inserted sequentially to the pCDH-CMV-MCS-EF1-Puro plasmid (System Biosciences) between the EcoRI and NotI sites (for Prdm16-FL and Prdm16-PRdeletion) and the XbaI and EcoRI sites (for NLS-3×FLAG).

To clone *shFlrt3*, annealed oligos were cloned into the HpaI and XhoI sites of the lentiviral vector pLL3.7, which contains a separate CMV promoter that drives the expression of dsRed. *Flrt3* shRNAs effectively suppressed co-expressed Flag-FLRT3 protein in HEK293T cells.

### Immunochemistry, BrdU and EdU labelling and confocal imaging

At designed stages, embryos or pups were perfused with PBS followed by 4% paraformaldehyde. The perfused brains were dissected, fixed overnight and sectioned coronally at 60 μm using a vibratome (Leica Microsystems, VT1200S). Immunostaining was carried out according to standard protocols as previously used (Dai et al., 2013a). Details of primary and secondary antibodies used are provided in Table S4.

BrdU and EdU (5-20 μg/g of body weight) were injected into the peritoneal cavity of pregnant mice. BrdU incorporation was measured by immunostaining using an antibody against rat-BrdU (Abcam) and mouse-BrdU (Developmental Studies Hybridoma Bank; DSHB). EdU incorporation was detected with the Click-iT assay (Invitrogen) according to the manufacturer's instructions. Imaging was carried out on a Zeiss confocal microscope. ZEN (Zeiss LSM800), ImageJ (National Institutes of Health) and Photoshop (Adobe) were used for analysis and quantification.

### *In situ* hybridization

The mouse brains at defined ages were dissected and fixed for 12 h in 4% PFA, dehydrated in 25% sucrose overnight, cryoprotected and embedded in O.C.T, and sectioned at 18 μm thickness on a Leica cryostat (CM3050s). RNA *in situ* hybridization was performed using digoxigenin-labeled riboprobes as previously described (He et al., 2018). Images were taken using a Leica DMLB microscope.

Quantification of *in situ* signal intensity was carried out using ImageJ/Fiji software. Areas at the labeled region (VZ/SVZ) and background region were selected and quantified for signal. The value was then normalized to area size.

### Quantification and statistical analysis

Cell numbers were manually counted in ImageJ/Fiji cell counter. The number of marker-positive cells in the control and KO mutant at P0 was determined by counting the average number of positive cells in three 80 µm-wide columns. The number of marker-positive cells in the control and KO or cKO mutants at E15.5 was determined by counting the number of positive cells in a 100 µm-wide column from layer IV to VI. The number of marker-positive cells in the control and cKO cortex at P15 was determined by counting the number of positive cells in one 250 µm-wide column within the whole cortex in two different areas (medial and dorsal lateral). For proliferation analysis at E15.5, the number of PAX6+, TBR2+, EdU+ and KI67+ cells was determined by counting the total number of positive cells in two 100 µm-wide columns in both VZ and SVZ. For proliferation analysis at E13.5, the number of PAX6+, TBR2+, EdU+ and KI67+ cells was determined by counting the total number in the 300 µm-wide cortex. The production of daughter neurons was reflected by the cell cycle exit through measuring the ratio of EdU+KI67-cells in total EdU+ cells. The number of BrdU+CTIP2+ or BrdU−CTIP2+ cells at P5 was determined by counting the number of positive cells in a 300 µm-wide column in layer II to layer V. For *in utero* electroporation followed by indirect neurogenesis analysis, the number of GFP+ and TBR2+ cells was determined by counting the total number of positive cells in one 250 µm-wide column in both the VZ and SVZ. For *in utero* electroporation followed by migration defect analysis, the number of GFP+ cells was determined by counting the total number of positive cells in one 300 µm-wide column in both white matter and cortex. For *in utero* electroporation followed by progenitor proliferation analysis, the number of GFP+ and EdU+ cells was determined by counting the total number of positive cells in one 250 µm-wide column in the SVZ. All data are presented as mean±s.d., and statistical significance was determined using a two-tailed unpaired Student's *t*-test.

### Neural stem cell culture and RT-qPCR

Control and mutant embryonic cortices were dissected and dissociated into single cell suspension and digested with Accutase (Sigma-Aldrich). Cells were maintained in proliferation media (Stemcell Technologies). Three control or three *Prdm16* mutant neural stem cell cultures were grown for 2 days before RNA extraction by use of TRIzol reagent (Invitrogen). Then, 4 μg of total RNA was further cleaned with Turbo DNase (Ambion) and used in reverse-transcription with RT master mix (Thermo Fisher Scientific). To ensure the absence of genomic DNA, control qPCR was performed on a mock-reverse-transcribed RNA sample. Primer sequences are listed in Table S4.

### Cell culture and luciferase assays

The neuroblastoma cell line Neuro-2A (N2A, ATCC^®^ CCL-131™) cells were cultured in 50% of DMEM (Gibco) containing 10% fetal calf serum and 50% of Opti-MEM serum reduced medium. For luciferase assays, transfections were performed in 96-well plate using FugeneHD transfection reagent (Promega). The following DNA combinations were used: 20 ng of *Fezf2*, *Cdkn1c*, *Flrt3* luciferase reporters or the pGL3 promoter vector, 100 ng of pCAGIG-Prdm16, pCAGIG-Prdm16PRdeletion, pCAGIG-Prdm16-VP64, pCAGIG-VP64 or pCAGIG. We used 2 ng of *Renilla* luciferase construct as an internal control. After 24 h incubation, transfected cells were lysed and luciferase activity was measured using Dual Luciferase Assays (Promega), and promoter activity was defined as the ratio between the firefly and *Renilla* luciferase activities.

For generating the cell lines that stably express control, PRDM16-FL or PRDM16-PRdeletion, lentiviral particles were first produced in 293T cells and then added to N2A cells for infection. The cells that stably expressed the corresponding constructs were selected and maintained in medium that contains puromycin. Two individual stable lines were generated for each of the constructs used in RT-qPCR analysis of the *Fezf2* gene.

### Western blotting

Western blots were used to examine shRNA knockdown efficiency. N2A cells were co-transfected with the constructs that express Flag-tagged FlRT3 or Flag-tagged CDKN1C and the corresponding shRNAs. After 24 h incubation, untransfected and transfected cells were harvested and lysed for protein extraction. Then the protein extract was run on 10% SDS PAGE gels. Proteins were then transferred to a PVDF membrane. The membranes were treated with blocking reagent (3% bovine serum albumin) for 1 h before incubated with the rabbit anti-RFP antibody (Abcam) overnight in a cold room. After several washes, the membranes were incubated with the secondary antibody (goat anti-rabbit immunoglobulins/HRP), washed again, and developed with the SuperSignal West Femto Maximum Sensitivity Substrate (Thermo Fisher Scientific, 34096). Images were collected using a Gel Doc XR+ Gel Documentation System (Bio-Rad) machine. The same membranes were then washed and incubated with the mouse Flag-m2 antibody (Sigma-Aldrich) overnight. On the second day, the membranes were washed, incubated with the secondary antibody (rabbit anti-mouse immunoglobulins/HRP), washed again, developed and exposed in the Gel Doc XR+ Gel Documentation System (Bio-Rad) machine. Details of the antibodies used are provided in Table S4.

### ChIP-seq analysis

In each replicate, three E13.5 control or Prdm16 KO mutant heads were pooled, fixed and lysed. ChIP was performed as previously described (Dai et al., 2013b). DNA libraries were made using the NEBNext Ultra™ II DNA Library Prep Kit and sequenced on the Illumina Hiseq2500 platform.

The replicated *Prdm16* KO (×3) and control (×3) ChIP-seq samples, after the adaptor trimming by Trimmomatic, were mapped to the University of California, Santa Cruz (UCSC) *Mus musculus* (mm10) genome assembly using Bowtie2 with the default parameters. The uniquely mapped reads (with mapping quality ≥20) were used for further analysis. The PRDM16 peaks were called by HOMER (v4.10) (Heinz et al., 2010). The peak replicate reproducibility was estimated by IDR, using the HOMER IDR pipeline (https://github.com/karmel/homer-idr). As suggested by the Encode IDR guideline that IDR requires to initially call peaks permissively for the replicates, we used a relatively relaxed parameter ‘-F 2 -fdr 0.3 -P 0.1 -L 3 -LP 0.1’ for the true/pseudo/pooled replicates by the HOMER peak calling. The final confidence peaks were determined by an IDR<5%. The peaks that were overlapped with mm10 blacklist were also removed. For comparisons, we re-analyzed the *Prdm16* control and cKO ChIP-seq public data (Baizabal et al., 2018; GSE111657) using the same HOMER IDR pipeline. All called ChIP-seq peaks are included in Table S2.

### RNA-seq differential expression analysis

Cortices of control and Prdm16 KO mutant E13.5 embryos were dissected for RNA extraction using TRIzol reagent (Invitrogen). RNA quality of three biological replicates was tested by Agilent Bioanalyzer. RNA-seq libraries were made using the Illumina Truseq Total RNA library Prep Kit LT. Sequencing was performed on the Illumina Hiseq2500 platform.

After trimming the adaptor sequences using Trimmomatic, we mapped RNA-seq reads from the replicated *Prdm16* wild type (×3) and mutant samples (×3) to the UCSC *Mus musculus* (mm10) genome assembly using HISAT2. We normalized RNA-seq by the ‘Relative Log Expression’ method implemented in the DESeq2 Bioconductor library (Love et al., 2014). Gene annotation was obtained from the iGenomes UCSC *Mus musculus* gene annotation. Differentially expressed mRNAs between *Prdm16* mutants versus wild type were identified, and FDR (Benjamini-Hochberg) was estimated using DESeq2. For comparisons, we re-analyzed the differential expression of *Prdm16* WT and cKO RNA-seq public data (Baizabal et al., 2018; GSE111660) using the same method as above. The genes with *P*-value ≤0.05 were considered to be differentially expressed. Differentially expressed genes are listed in Table S1.

### ATAC-seq analysis

The ATAC-seq libraries were made according to the published method (Buenrostro et al., 2013) and using the Illumina Nextera DNA library kit. In brief, cortices were dissected from three control and three *Prdm16* KO E13.5 brains. Tn5 enzyme reaction was performed at 37 C for 30 min, followed by DNA purification. Eleven cycles of PCR amplification were performed using barcoded adaptors and primers on a purified DNA template. Libraries were purified and pooled before sequencing with the Illumina Next-seq platform. The replicated *Prdm16* KO (×3) and control (×3) ATAC-seq samples, after the adaptor trimming by Trimmomatic, were mapped to the UCSC *Mus musculus* (mm10) genome assembly using Bowtie2 with the default parameters. The high quality and uniquely mapped reads (with mapping quality ≥20) were used for further analysis. ATAC-seq differential expression analysis between *Prdm16* mutants and wild types on the *Prdm16*-bound ChIP-seq peaks was performed using the Limma R package. The ATAC-seq peak calling was performed by HOMER using the ‘broad peak’ option with parameters ‘-region -size 1000 -minDist 2500’, separately for the mutant and wild type. To compare active enhancers between E13.5 and E15.5, we further re-analyzed the publicly available histone mark H3K27ac and PRDM16 ChIP-seq data at E15.5. We called the peaks against input using ‘narrow peak’ option in HOMER with the default parameters.

### Gene set enrichment testing

To test whether a set of genes is significantly changed amongst the differentially expressed genes from *Prdm16* wild type and mutant RNA-seq data, we used gene set testing function ‘camera’ and ‘mroast’ in the Limma R package (Ritchie et al., 2015). We used ‘camera’, a ranking-based gene set test accounting for inter-gene correlation, to test whether the layer markers were significantly changed as a set. We used ‘mroast’ (number of rotations=1000), a self-contained gene set test, to test whether the majority of the genes amongst PRDM16 targets were significantly up- or downregulated. We also used ‘mroast’ to test which GO terms and Reactome pathways were significantly up- or downregulated in *Prdm16* mutant versus wild type.

### scRNA-seq analysis

To gain insights into cell types of the *Prdm16* targets, we reanalyzed the murine cortical time-series scRNA-seq data (Yuzwa et al., 2017). We employed the Bioconductor scRNA-seq analysis workflow for droplet-based protocols (Lun et al., 2016). (1) The cortical cells (the cells expressing Emx) were selected for the analysis. The low quality cells were first removed if they were 3 median absolute deviation (MAD) lower than the median library size or if they were 3 MAD lower the median gene expression or if they were 4 MAD higher than the median mitochondrial reads. (2) We used the deconvolution approach, a method to handle high zeros in scRNA-seq, to compute size factors for cells for normalization. (3) The cells were constructed into graphs by creating a shared nearest neighbor graph and clustered by Walktrap algorithm. (4) We manually assigned cell types to the identified clusters in each stage using the known neuron and layer markers. For each cell, the sum of (log2) reads for all *Prdm16* targets was calculated. The number of cells with >200 sum of reads divided by the total number of cells in each cluster were calculated to give the percentage of cells that *Prdm16* targets expressed in Fig. 6.

To identify differentially expressed genes between E13.5 and E15.5, RG cells were extracted from E13.5 and E15.5 RG clusters and differential expression analysis was performed using the edgeRQLF R package. The genes with FDR≤0.2 and FC>1.4-fold were considered to be significantly differentially expressed between E13.5 and E15.5, and are listed in Table S3.

## Supplementary Material

Supplementary information

Reviewer comments

## References

[DEV194670C1] Aguilo, F., Avagyan, S., Labar, A., Sevilla, A., Lee, D. F., Kumar, P., Lemischka, I. R., Zhou, B. Y. and Snoeck, H. W. (2011). Prdm16 is a physiologic regulator of hematopoietic stem cells. *Blood* 117, 5057-5066. 10.1182/blood-2010-08-300145 21343612 PMC3109532

[DEV194670C2] Angevine, J. B., Jr and Sidman, R. L. (1961). Autoradiographic study of cell migration during histogenesis of cerebral cortex in the mouse. *Nature* 192, 766-768. 10.1038/192766b0 17533671

[DEV194670C3] Anthony, T. E., Klein, C., Fishell, G. and Heintz, N. (2004). Radial glia serve as neuronal progenitors in all regions of the central nervous system. *Neuron* 41, 881-890. 10.1016/S0896-6273(04)00140-0 15046721

[DEV194670C4] Arndt, A. K., Schafer, S., Drenckhahn, J. D., Sabeh, M. K., Plovie, E. R., Caliebe, A., Klopocki, E., Musso, G., Werdich, A. A., Kalwa, H. et al. (2013). Fine mapping of the 1p36 deletion syndrome identifies mutation of PRDM16 as a cause of cardiomyopathy. *Am. J. Hum. Genet.* 93, 67-77. 10.1016/j.ajhg.2013.05.015 23768516 PMC3710750

[DEV194670C5] Baizabal, J.-M., Mistry, M., García, M. T., Gómez, N., Olukoya, O., Tran, D., Johnson, M. B., Walsh, C. A. and Harwell, C. C. (2018). The epigenetic state of PRDM16-regulated enhancers in radial glia controls cortical neuron position. *Neuron* 98, 945-962.e8. 10.1016/j.neuron.2018.04.033 29779941 PMC6667181

[DEV194670C6] Bjork, B. C., Fujiwara, Y., Davis, S. W., Qiu, H., Saunders, T. L., Sandy, P., Orkin, S., Camper, S. A. and Beier, D. R. (2010a). A transient transgenic RNAi strategy for rapid characterization of gene function during embryonic development. *PLoS One* 5, e14375. 10.1371/journal.pone.0014375 21179568 PMC3002952

[DEV194670C7] Bjork, B. C., Turbe-Doan, A., Prysak, M., Herron, B. J. and Beier, D. R. (2010b). Prdm16 is required for normal palatogenesis in mice. *Hum. Mol. Genet.* 19, 774-789. 10.1093/hmg/ddp543 20007998 PMC2816611

[DEV194670C8] Buenrostro, J. D., Giresi, P. G., Zaba, L. C., Chang, H. Y. and Greenleaf, W. J. (2013). Transposition of native chromatin for fast and sensitive epigenomic profiling of open chromatin, DNA-binding proteins and nucleosome position. *Nat. Methods* 10, 1213-1218. 10.1038/nmeth.2688 24097267 PMC3959825

[DEV194670C9] Chenn, A. and Walsh, C. A. (2002). Regulation of cerebral cortical size by control of cell cycle exit in neural precursors. *Science* 297, 365-369. 10.1126/science.1074192 12130776

[DEV194670C10] Chi, J. and Cohen, P. (2016). The multifaceted roles of PRDM16: adipose biology and beyond. *Trends Endocrinol. Metab.* 27, 11-23. 10.1016/j.tem.2015.11.005 26688472

[DEV194670C11] Chui, A., Zhang, Q., Dai, Q. and Shi, S. H. (2020). Oxidative stress regulates progenitor behavior and cortical neurogenesis. *Development* 147, dev184150. 10.1242/dev.184150 32041791 PMC7075051

[DEV194670C12] Chuikov, S., Levi, B. P., Smith, M. L. and Morrison, S. J. (2010). Prdm16 promotes stem cell maintenance in multiple tissues, partly by regulating oxidative stress. *Nat. Cell Biol.* 12, 999-1006. 10.1038/ncb2101 20835244 PMC2948585

[DEV194670C13] Cohen, P., Levy, J. D., Zhang, Y., Frontini, A., Kolodin, D. P., Svensson, K. J., Lo, J. C., Zeng, X., Ye, L., Khandekar, M. J. et al. (2014). Ablation of PRDM16 and beige adipose causes metabolic dysfunction and a subcutaneous to visceral fat switch. *Cell* 156, 304-316. 10.1016/j.cell.2013.12.021 24439384 PMC3922400

[DEV194670C14] Corrigan, D. J., Luchsinger, L. L., Justino De Almeida, M., Williams, L. J., Strikoudis, A. and Snoeck, H. W. (2018). PRDM16 isoforms differentially regulate normal and leukemic hematopoiesis and inflammatory gene signature. *J. Clin. Invest.* 128, 3250-3264. 10.1172/JCI99862 29878897 PMC6063481

[DEV194670C15] Dai, Q., Andreu-Agullo, C., Insolera, R., Wong, L. C., Shi, S.-H. and Lai, E. C. (2013a). BEND6 is a nuclear antagonist of Notch signaling during self-renewal of neural stem cells. *Development* 140, 1892-1902. 10.1242/dev.087502 23571214 PMC3631965

[DEV194670C16] Dai, Q., Ren, A., Westholm, J. O., Serganov, A. A., Patel, D. J. and Lai, E. C. (2013b). The BEN domain is a novel sequence-specific DNA-binding domain conserved in neural transcriptional repressors. *Genes Dev.* 27, 602-614. 10.1101/gad.213314.113 23468431 PMC3613608

[DEV194670C17] Daugherty, A. C., Yeo, R. W., Buenrostro, J. D., Greenleaf, W. J., Kundaje, A. and Brunet, A. (2017). Chromatin accessibility dynamics reveal novel functional enhancers in C. elegans. *Genome Res.* 27, 2096-2107. 10.1101/gr.226233.117 29141961 PMC5741055

[DEV194670C18] Dennis, D. J., Wilkinson, G., Li, S., Dixit, R., Adnani, L., Balakrishnan, A., Han, S., Kovach, C., Gruenig, N., Kurrasch, D. M. et al. (2017). Neurog2 and Ascl1 together regulate a postmitotic derepression circuit to govern laminar fate specification in the murine neocortex. *Proc. Natl. Acad. Sci. USA* 114, E4934-E4943. 10.1073/pnas.1701495114 28584103 PMC5488939

[DEV194670C19] Desai, A. R. and Mcconnell, S. K. (2000). Progressive restriction in fate potential by neural progenitors during cerebral cortical development. *Development* 127, 2863-2872. 10851131 10.1242/dev.127.13.2863

[DEV194670C20] Eroglu, E., Burkard, T. R., Jiang, Y., Saini, N., Homem, C. C., Reichert, H. and Knoblich, J. A. (2014). SWI/SNF complex prevents lineage reversion and induces temporal patterning in neural stem cells. *Cell* 156, 1259-1273. 10.1016/j.cell.2014.01.053 24630726

[DEV194670C21] Frantz, G. D. and Mcconnell, S. K. (1996). Restriction of late cerebral cortical progenitors to an upper-layer fate. *Neuron* 17, 55-61. 10.1016/S0896-6273(00)80280-9 8755478

[DEV194670C22] Fukumitsu, H., Ohtsuka, M., Murai, R., Nakamura, H., Itoh, K. and Furukawa, S. (2006). Brain-derived neurotrophic factor participates in determination of neuronal laminar fate in the developing mouse cerebral cortex. *J. Neurosci.* 26, 13218-13230. 10.1523/JNEUROSCI.4251-06.2006 17182772 PMC6675008

[DEV194670C23] Gao, P., Postiglione, M. P., Krieger, T. G., Hernandez, L., Wang, C., Han, Z., Streicher, C., Papusheva, E., Insolera, R., Chugh, K. et al. (2014). Deterministic progenitor behavior and unitary production of neurons in the neocortex. *Cell* 159, 775-788. 10.1016/j.cell.2014.10.027 25417155 PMC4225456

[DEV194670C24] Gaspard, N., Bouschet, T., Hourez, R., Dimidschstein, J., Naeije, G., Van Den Ameele, J., Espuny-Camacho, I., Herpoel, A., Passante, L., Schiffmann, S. N. et al. (2008). An intrinsic mechanism of corticogenesis from embryonic stem cells. *Nature* 455, 351-357. 10.1038/nature07287 18716623

[DEV194670C25] Ge, W., He, F., Kim, K. J., Blanchi, B., Coskun, V., Nguyen, L., Wu, X., Zhao, J., Heng, J. I., Martinowich, K. et al. (2006). Coupling of cell migration with neurogenesis by proneural bHLH factors. *Proc. Natl. Acad. Sci. USA* 103, 1319-1324. 10.1073/pnas.0510419103 16432194 PMC1345712

[DEV194670C26] Gorski, J. A., Talley, T., Qiu, M., Puelles, L., Rubenstein, J. L. and Jones, K. R. (2002). Cortical excitatory neurons and glia, but not GABAergic neurons, are produced in the Emx1-expressing lineage. *J. Neurosci.* 22, 6309-6314. 10.1523/JNEUROSCI.22-15-06309.2002 12151506 PMC6758181

[DEV194670C27] Greig, L. C., Woodworth, M. B., Galazo, M. J., Padmanabhan, H. and Macklis, J. D. (2013). Molecular logic of neocortical projection neuron specification, development and diversity. *Nat. Rev. Neurosci.* 14, 755-769. 10.1038/nrn3586 24105342 PMC3876965

[DEV194670C71] He, L., Yu, K., Lu, F., Wang, J., Wu, L. N., Zhao, C., Li, Q., Zhou, X., Liu, H., Mu, D. et al. (2018). Transcriptional regulator ZEB2 is essential for Bergmann glia development. *J. Neurosci.* 38, 1575-1587. 10.1523/JNEUROSCI.2674-17.2018 29326173 PMC5815355

[DEV194670C28] Heinz, S., Benner, C., Spann, N., Bertolino, E., Lin, Y. C., Laslo, P., Cheng, J. X., Murre, C., Singh, H. and Glass, C. K. (2010). Simple combinations of lineage-determining transcription factors prime cis-regulatory elements required for macrophage and B cell identities. *Mol. Cell* 38, 576-589. 10.1016/j.molcel.2010.05.004 20513432 PMC2898526

[DEV194670C29] Horn, K. H., Warner, D. R., Pisano, M. and Greene, R. M. (2011). PRDM16 expression in the developing mouse embryo. *Acta Histochem.* 113, 150-155. 10.1016/j.acthis.2009.09.006 19853285 PMC2891916

[DEV194670C30] Hsu, L. C.-L., Nam, S., Cui, Y., Chang, C.-P., Wang, C.-F., Kuo, H.-C., Touboul, J. D. and Chou, S.-J. (2015). Lhx2 regulates the timing of beta-catenin-dependent cortical neurogenesis. *Proc. Natl. Acad. Sci. USA* 112, 12199-12204. 10.1073/pnas.1507145112 26371318 PMC4593128

[DEV194670C31] Imayoshi, I., Shimogori, T., Ohtsuka, T. and Kageyama, R. (2008). Hes genes and neurogenin regulate non-neural versus neural fate specification in the dorsal telencephalic midline. *Development* 135, 2531-2541. 10.1242/dev.021535 18579678

[DEV194670C32] Inoue, M., Iwai, R., Tabata, H., Konno, D., Komabayashi-Suzuki, M., Watanabe, C., Iwanari, H., Mochizuki, Y., Hamakubo, T., Matsuzaki, F. et al. (2017). Prdm16 is crucial for progression of the multipolar phase during neural differentiation of the developing neocortex. *Development* 144, 385-399. 10.1242/dev.136382 27993981

[DEV194670C33] Johansson, P. A. (2014). The choroid plexuses and their impact on developmental neurogenesis. *Front. Neurosci.* 8, 340. 10.3389/fnins.2014.00340 25386116 PMC4208491

[DEV194670C34] Johansson, P. A., Irmler, M., Acampora, D., Beckers, J., Simeone, A. and Gotz, M. (2013). The transcription factor Otx2 regulates choroid plexus development and function. *Development* 140, 1055-1066. 10.1242/dev.090860 23364326

[DEV194670C35] Kajimura, S., Seale, P., Tomaru, T., Erdjument-Bromage, H., Cooper, M. P., Ruas, J. L., Chin, S., Tempst, P., Lazar, M. A. and Spiegelman, B. M. (2008). Regulation of the brown and white fat gene programs through a PRDM16/CtBP transcriptional complex. *Genes Dev.* 22, 1397-1409. 10.1101/gad.1666108 18483224 PMC2377193

[DEV194670C36] Kriegstein, A. and Alvarez-Buylla, A. (2009). The glial nature of embryonic and adult neural stem cells. *Annu. Rev. Neurosci.* 32, 149-184. 10.1146/annurev.neuro.051508.135600 19555289 PMC3086722

[DEV194670C37] Kwan, K. Y., Sestan, N. and Anton, E. S. (2012). Transcriptional co-regulation of neuronal migration and laminar identity in the neocortex. *Development* 139, 1535-1546. 10.1242/dev.069963 22492350 PMC3317962

[DEV194670C38] Lehtinen, M. K., Bjornsson, C. S., Dymecki, S. M., Gilbertson, R. J., Holtzman, D. M. and Monuki, E. S. (2013). The choroid plexus and cerebrospinal fluid: emerging roles in development, disease, and therapy. *J. Neurosci.* 33, 17553-17559. 10.1523/JNEUROSCI.3258-13.2013 24198345 PMC3818536

[DEV194670C39] Lehtinen, M. K., Zappaterra, M. W., Chen, X., Yang, Y. J., Hill, A. D., Lun, M., Maynard, T., Gonzalez, D., Kim, S., Ye, P. et al. (2011). The cerebrospinal fluid provides a proliferative niche for neural progenitor cells. *Neuron* 69, 893-905. 10.1016/j.neuron.2011.01.023 21382550 PMC3085909

[DEV194670C40] Love, M. I., Huber, W. and Anders, S. (2014). Moderated estimation of fold change and dispersion for RNA-seq data with DESeq2. *Genome Biol.* 15, 550. 10.1186/s13059-014-0550-8 25516281 PMC4302049

[DEV194670C41] Lun, A. T., Mccarthy, D. J. and Marioni, J. C. (2016). A step-by-step workflow for low-level analysis of single-cell RNA-seq data with Bioconductor. *F1000Res* 5, 2122. 10.12688/f1000research.9501.2 27909575 PMC5112579

[DEV194670C42] Mairet-Coello, G., Tury, A., Van Buskirk, E., Robinson, K., Genestine, M. and Dicicco-Bloom, E. (2012). p57(KIP2) regulates radial glia and intermediate precursor cell cycle dynamics and lower layer neurogenesis in developing cerebral cortex. *Development* 139, 475-487. 10.1242/dev.067314 22223678 PMC3252351

[DEV194670C43] Mcconnell, S. K. and Kaznowski, C. E. (1991). Cell cycle dependence of laminar determination in developing neocortex. *Science* 254, 282-285. 10.1126/science.1925583 1925583

[DEV194670C44] Mcevilly, R. J., De Diaz, M. O., Schonemann, M. D., Hooshmand, F. and Rosenfeld, M. G. (2002). Transcriptional regulation of cortical neuron migration by POU domain factors. *Science* 295, 1528-1532. 10.1126/science.1067132 11859196

[DEV194670C45] Mckenna, W. L., Ortiz-Londono, C. F., Mathew, T. K., Hoang, K., Katzman, S. and Chen, B. (2015). Mutual regulation between Satb2 and Fezf2 promotes subcerebral projection neuron identity in the developing cerebral cortex. *Proc. Natl. Acad. Sci. USA* 112, 11702-11707. 10.1073/pnas.1504144112 26324926 PMC4577201

[DEV194670C46] Molyneaux, B. J., Arlotta, P., Hirata, T., Hibi, M. and Macklis, J. D. (2005). Fezl is required for the birth and specification of corticospinal motor neurons. *Neuron* 47, 817-831. 10.1016/j.neuron.2005.08.030 16157277

[DEV194670C47] Molyneaux, B. J., Arlotta, P., Menezes, J. R. and Macklis, J. D. (2007). Neuronal subtype specification in the cerebral cortex. *Nat. Rev. Neurosci.* 8, 427-437. 10.1038/nrn2151 17514196

[DEV194670C48] Nishikata, I., Sasaki, H., Iga, M., Tateno, Y., Imayoshi, S., Asou, N., Nakamura, T. and Morishita, K. (2003). A novel EVI1 gene family, MEL1, lacking a PR domain (MEL1S) is expressed mainly in t(1;3)(p36;q21)-positive AML and blocks G-CSF-induced myeloid differentiation. *Blood* 102, 3323-3332. 10.1182/blood-2002-12-3944 12816872

[DEV194670C49] Noctor, S. C., Martínez-Cerdeño, V., Ivic, L. and Kriegstein, A. R. (2004). Cortical neurons arise in symmetric and asymmetric division zones and migrate through specific phases. *Nat. Neurosci.* 7, 136-144. 10.1038/nn1172 14703572

[DEV194670C50] Okano, H. and Temple, S. (2009). Cell types to order: temporal specification of CNS stem cells. *Curr. Opin. Neurobiol.* 19, 112-119. 10.1016/j.conb.2009.04.003 19427192

[DEV194670C51] Pinheiro, I., Margueron, R., Shukeir, N., Eisold, M., Fritzsch, C., Richter, F. M., Mittler, G., Genoud, C., Goyama, S., Kurokawa, M. et al. (2012). Prdm3 and Prdm16 are H3K9me1 methyltransferases required for mammalian heterochromatin integrity. *Cell* 150, 948-960. 10.1016/j.cell.2012.06.048 22939622

[DEV194670C52] Ritchie, M. E., Phipson, B., Wu, D., Hu, Y., Law, C. W., Shi, W. and Smyth, G. K. (2015). limma powers differential expression analyses for RNA-sequencing and microarray studies. *Nucleic Acids Res.* 43, e47. 10.1093/nar/gkv007 25605792 PMC4402510

[DEV194670C53] Rubinson, D. A., Dillon, C. P., Kwiatkowski, A. V., Sievers, C., Yang, L., Kopinja, J., Rooney, D. L., Zhang, M., Ihrig, M. M., Mcmanus, M. T. et al. (2003). A lentivirus-based system to functionally silence genes in primary mammalian cells, stem cells and transgenic mice by RNA interference. *Nat. Genet.* 33, 401-406. 10.1038/ng1117 12590264

[DEV194670C54] Saito, T. (2006). In vivo electroporation in the embryonic mouse central nervous system. *Nat. Protoc.* 1, 1552-1558. 10.1038/nprot.2006.276 17406448

[DEV194670C55] Saito, T. and Nakatsuji, N. (2001). Efficient gene transfer into the embryonic mouse brain using in vivo electroporation. *Dev. Biol.* 240, 237-246. 10.1006/dbio.2001.0439 11784059

[DEV194670C56] Shen, Q., Wang, Y., Dimos, J. T., Fasano, C. A., Phoenix, T. N., Lemischka, I. R., Ivanova, N. B., Stifani, S., Morrisey, E. E. and Temple, S. (2006). The timing of cortical neurogenesis is encoded within lineages of individual progenitor cells. *Nat. Neurosci.* 9, 743-751. 10.1038/nn1694 16680166

[DEV194670C57] Shim, S., Kwan, K. Y., Li, M., Lefebvre, V. and Sestan, N. (2012). Cis-regulatory control of corticospinal system development and evolution. *Nature* 486, 74-79. 10.1038/nature11094 22678282 PMC3375921

[DEV194670C58] Shimada, I. S., Acar, M., Burgess, R. J., Zhao, Z. and Morrison, S. J. (2017). Prdm16 is required for the maintenance of neural stem cells in the postnatal forebrain and their differentiation into ependymal cells. *Genes Dev.* 31, 1134-1146. 10.1101/gad.291773.116 28698301 PMC5538436

[DEV194670C59] Srinivasan, K., Leone, D. P., Bateson, R. K., Dobreva, G., Kohwi, Y., Kohwi-Shigematsu, T., Grosschedl, R. and Mcconnell, S. K. (2012). A network of genetic repression and derepression specifies projection fates in the developing neocortex. *Proc. Natl. Acad. Sci. USA* 109, 19071-19078. 10.1073/pnas.1216793109 23144223 PMC3511157

[DEV194670C60] Strassman, A., Schnutgen, F., Dai, Q., Jones, J. C., Gomez, A. C., Pitstick, L., Holton, N. E., Moskal, R., Leslie, E. R., Von Melchner, H. et al. (2017). Generation of a multipurpose Prdm16 mouse allele by targeted gene trapping. *Dis. Model. Mech.* 10, 909-922. 10.1242/dmm.029561 28424158 PMC5536910

[DEV194670C61] Sugitani, Y., Nakai, S., Minowa, O., Nishi, M., Jishage, K., Kawano, H., Mori, K., Ogawa, M. and Noda, T. (2002). Brn-1 and Brn-2 share crucial roles in the production and positioning of mouse neocortical neurons. *Genes Dev.* 16, 1760-1765. 10.1101/gad.978002 12130536 PMC186401

[DEV194670C62] Takahashi, T., Nowakowski, R. S. and Caviness, V. S. Jr. (1995). The cell cycle of the pseudostratified ventricular epithelium of the embryonic murine cerebral wall. *J. Neurosci.* 15, 6046-6057. 10.1523/JNEUROSCI.15-09-06046.1995 7666188 PMC6577667

[DEV194670C63] Telley, L., Agirman, G., Prados, J., Amberg, N., Fievre, S., Oberst, P., Bartolini, G., Vitali, I., Cadilhac, C., Hippenmeyer, S. et al. (2019). Temporal patterning of apical progenitors and their daughter neurons in the developing neocortex. *Science* 364, eaav2522. 10.1126/science.aav2522 31073041

[DEV194670C64] Vandenberg, R. J. and Ryan, R. M. (2013). Mechanisms of glutamate transport. *Physiol. Rev.* 93, 1621-1657. 10.1152/physrev.00007.2013 24137018

[DEV194670C65] Vitali, I., Fievre, S., Telley, L., Oberst, P., Bariselli, S., Frangeul, L., Baumann, N., Mcmahon, J. J., Klingler, E., Bocchi, R. et al. (2018). Progenitor hyperpolarization regulates the sequential generation of neuronal subtypes in the developing neocortex. *Cell* 174, 1264-1276.e15. 10.1016/j.cell.2018.06.036 30057116 PMC6545245

[DEV194670C66] Yamagishi, S., Hampel, F., Hata, K., Del Toro, D., Schwark, M., Kvachnina, E., Bastmeyer, M., Yamashita, T., Tarabykin, V., Klein, R. et al. (2011). FLRT2 and FLRT3 act as repulsive guidance cues for Unc5-positive neurons. *EMBO J.* 30, 2920-2933. 10.1038/emboj.2011.189 21673655 PMC3160250

[DEV194670C67] Yoon, K. J., Ringeling, F. R., Vissers, C., Jacob, F., Pokrass, M., Jimenez-Cyrus, D., Su, Y., Kim, N. S., Zhu, Y., Zheng, L. et al. (2017). Temporal control of mammalian cortical neurogenesis by m(6)A methylation. *Cell* 171, 877-889.e17. 10.1016/j.cell.2017.09.003 28965759 PMC5679435

[DEV194670C68] Yuzwa, S. A., Borrett, M. J., Innes, B. T., Voronova, A., Ketela, T., Kaplan, D. R., Bader, G. D. and Miller, F. D. (2017). Developmental emergence of adult neural stem cells as revealed by single-cell transcriptional profiling. *Cell Rep* 21, 3970-3986. 10.1016/j.celrep.2017.12.017 29281841

[DEV194670C69] Zahr, S. K., Yang, G., Kazan, H., Borrett, M. J., Yuzwa, S. A., Voronova, A., Kaplan, D. R. and Miller, F. D. (2018). A translational repression complex in developing mammalian neural stem cells that regulates neuronal specification. *Neuron* 97, 520-537.e6. 10.1016/j.neuron.2017.12.045 29395907

[DEV194670C70] Zhou, B., Wang, J., Lee, S. Y., Xiong, J., Bhanu, N., Guo, Q., Ma, P., Sun, Y., Rao, R. C., Garcia, B. A. et al. (2016). PRDM16 suppresses MLL1r leukemia via intrinsic histone methyltransferase activity. *Mol. Cell* 62, 222-236. 10.1016/j.molcel.2016.03.010 27151440 PMC5061593

