# Supplementary material for: PRDM16 regulates a temporal transcriptional program to promote progression of cortical neural progenitors
Source: Development. 2021 Mar 17;148(6):dev194670. doi: 10.1242/dev.194670 (PMC7990860; doi:10.1242/dev.194670)
Supplement: Supplementary information [file develop-148-194670-s1.pdf]

Table S1. All genes in FB RNA-seq data.

Click here to Download Table S1

Table S2. All genes from PRDM16 ChIP-seq calls using E13.5 whole head ChIP-seq (this study) and E15.5 cortex (Baizabal et al. 2018)

Click here to Download Table S2

Table S3. Differentially expressed genes between the E13.5 and E15.5 RG clusters [re-analyzed from (Yuzwa et al. 2017)]

Click here to Download Table S3

Table S4. Reagents and oligonucleotide sequences.

Click here to Download Table S4

References:

Baizabal JM, Mistry M, Garcia MT, Gomez N, Olukoya O, Tran D, Johnson MB, Walsh CA, Harwell CC. 2018. The Epigenetic State of PRDM16-Regulated Enhancers in Radial Glia Controls Cortical Neuron Position. *Neuron* **98**: 945-962 e948.

Yuzwa SA, Borrett MJ, Innes BT, Voronova A, Ketela T, Kaplan DR, Bader GD, Miller FD. 2017. Developmental Emergence of Adult Neural Stem Cells as Revealed by Single-Cell Transcriptional Profiling. *Cell Rep* **21**: 3970-3986.

He_Suppl.Fig. 1

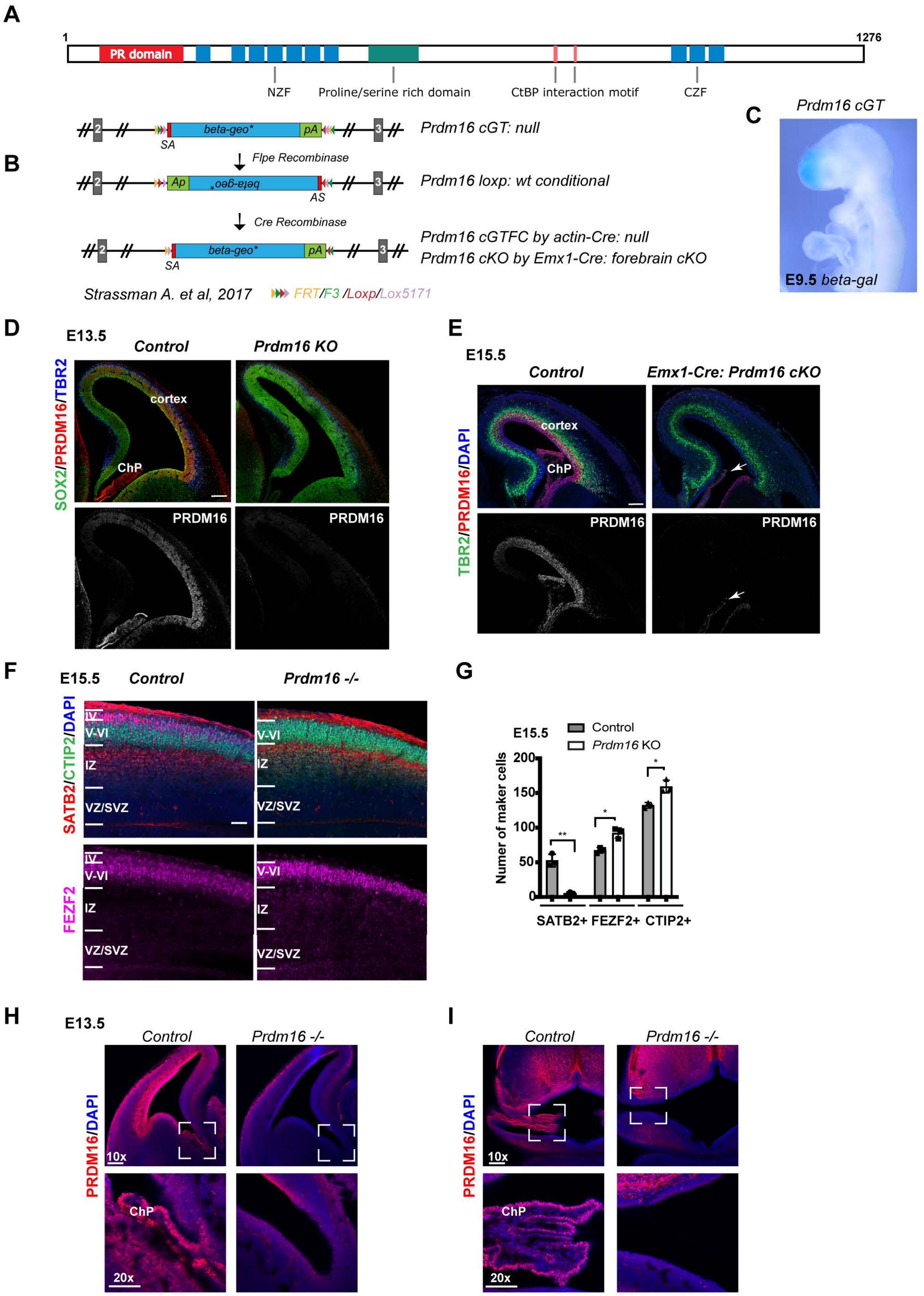

Development • Supplementary information

Figure S1: The PRDM16 protein and the gene-trap lines for generating null and conditional knockout animals

(**A**) Schematic of the largest isoform of the PRDM16 protein shows the functional domains of PRDM16. (**B**) Schematics of the gene-trap lines. (**C**) X-gal staining of a E9.5 embryo with the gene-trap (*Prdm16*^{cGT}) indicates that *Prdm16* is expressed at E9.5. (**D**) Coronal section slices of control or *Prdm16*^{cGT} KO mutant, stained with PRDM16 in red, SOX2 in green and TBR2 in blue. The PRDM16 staining is lost in the entire brain. Scale bar: 100 µm. (**E**) Coronal section slices of control or *Emx1*^{IREScre}-mediated *Prdm16* conditional mutant (*Prdm16* cKO), stained with PRDM16 in red, TBR2 in green and the nuclei marker DAPI in blue. Prdm16 staining is mostly lost in the forebrain but remained in the ventral telencephalon and the ChP. White arrows indicate the presence of the ChP in the mutant. (**F**) Images of E15.5 *Emx1-Cre::Prdm16* cKO cortices stained with antibodies against SATB2 in red, FEZF2 in magenta and CTIP2 in green. (**G**) Quantification of the marker+ cells in 100 µm column across the cortex of E15.5 (n=3). Scale bar: 50 µm. (**H-I**) Images of E13.5 cortices stained with the PRDM16 antibody and DAPI, showing high expression of PRDM16 in the ChP in the control but absent in *Prdm16* null mutant in the lateral ventricle (I), and in the 3rd ventricle (J). Lower panels are higher magnification images of the white square area of the upper panels. Scale bar: 50 µm. All data are shown as mean +/- SD; *p<0.05; **p<0.01; ***p<0.001.

Development • Supplementary information

He_Suppl.Fig. 2

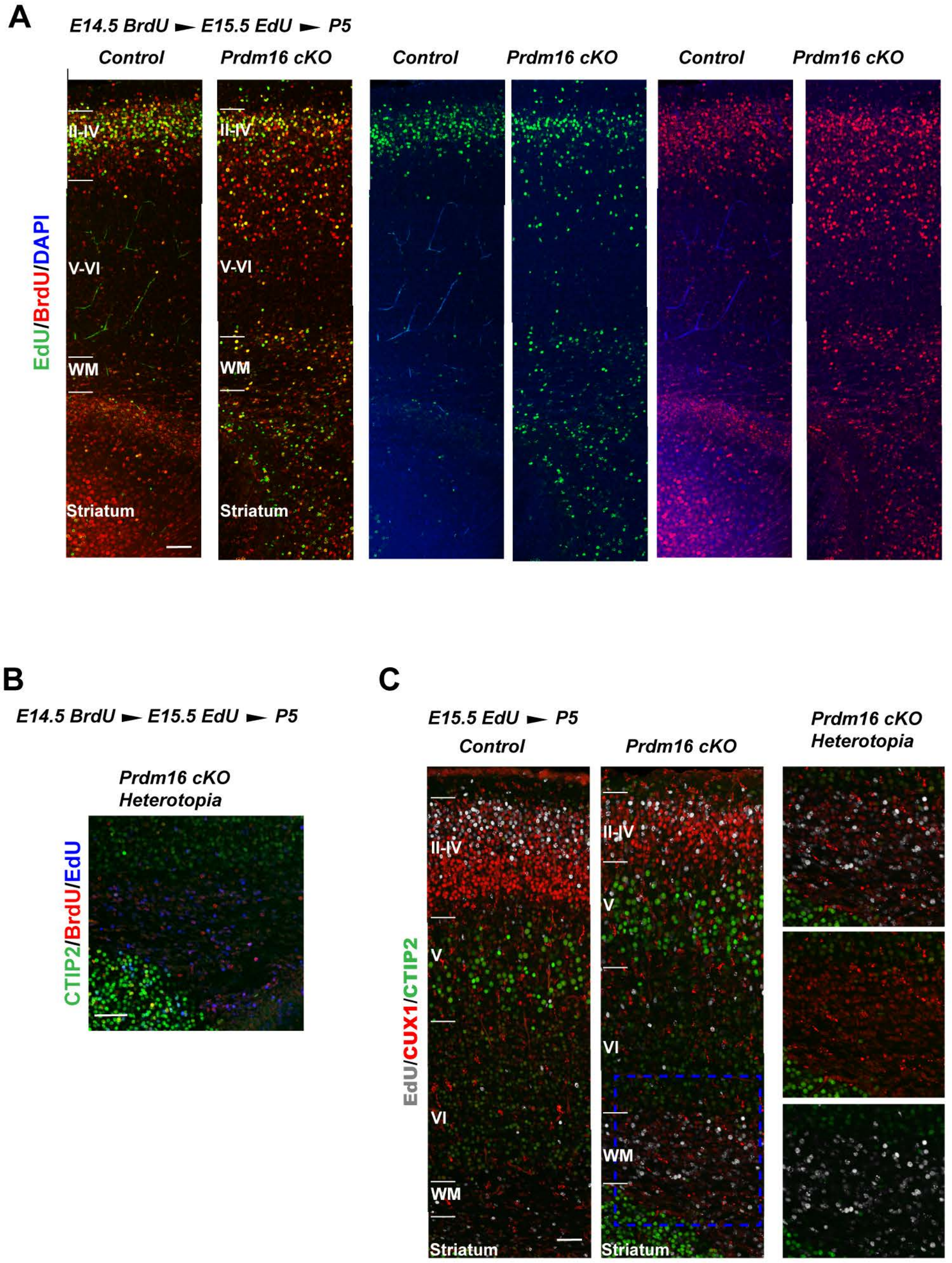

Figure S2: ***Prdm16*** **forebrain-specific knockout animals display neuronal migration defect.** **(A)** Coronal sections of P5 control and cKO brains, stained with Brdu in red, Edu in green and the nuclei marker DAPI in blue. The cells labeled with Brdu or Edu were retained in the deep layer and in the heterotopia tissue in mutant cortex. **(B)** The heterotopia area in the cKO section, stained with CTIP2 in green, Brdu in red and Edu in blue, showing lack of CTIP2+ and Brdu+ or Edu+ double positive cells in heterotopia. **(C)** Coronal sections of control and cKO brains, stained with CUX1 in red, Edu in gray and CTIP2 in green. All of Edu+ cells are positive for CUX1. **B-C** together shows failure of migration is in the neurons co-stained with upper-layer marker CUX1 but not with mid-layer marker CTIP2.

Development • Supplementary information

He_Suppl.Fig. 3

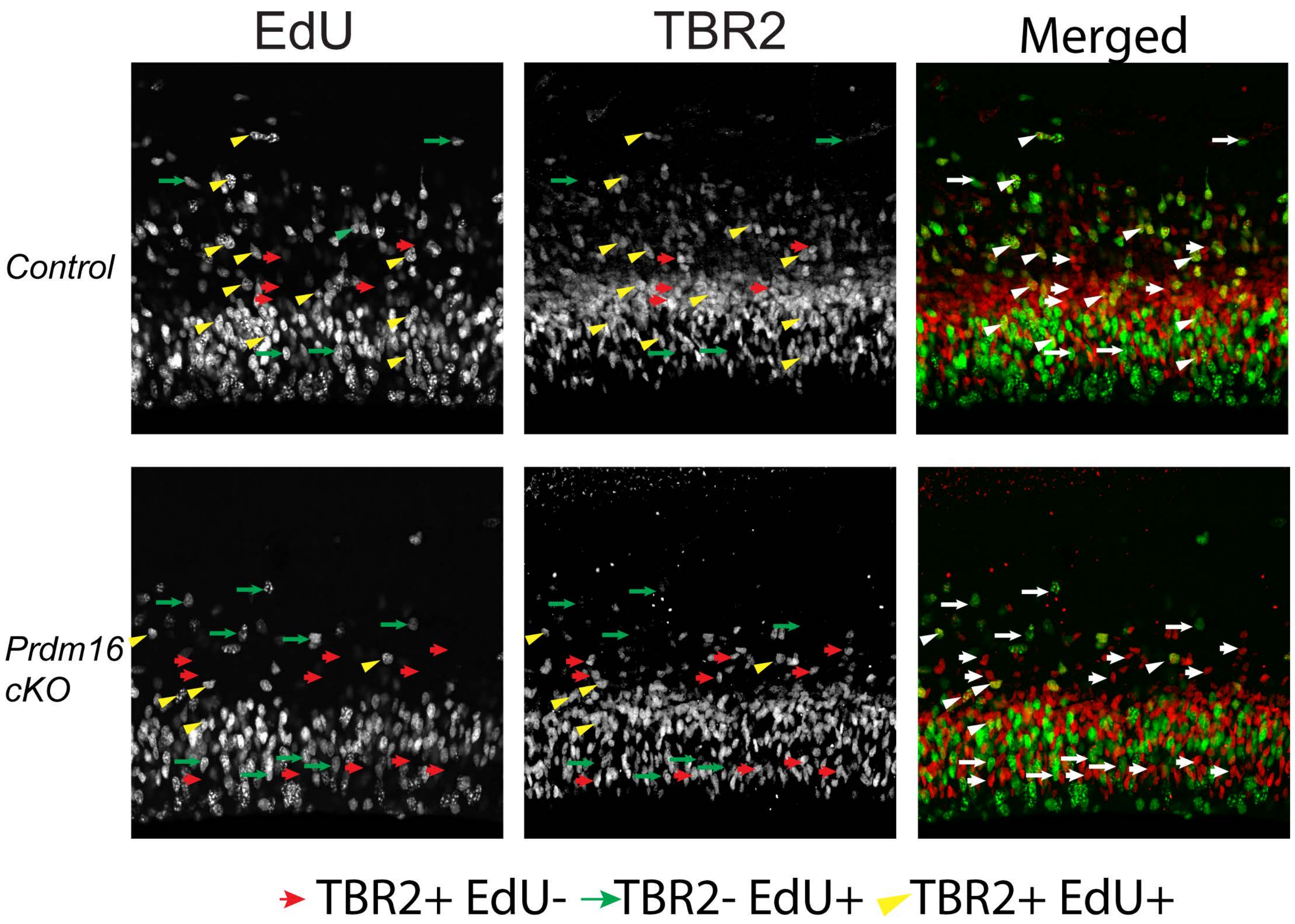

Figure S3: Single channel images of Figure 3H.

The arrowheads represent TBR2+EdU+ double positive cells, the long green and white arrows indicates TBR2 negative but EdU positive cells, and the short red and white arrows indicates TBR2 positive but EdU negative cells.

Development • Supplementary information

He_Suppl.Fig. 4

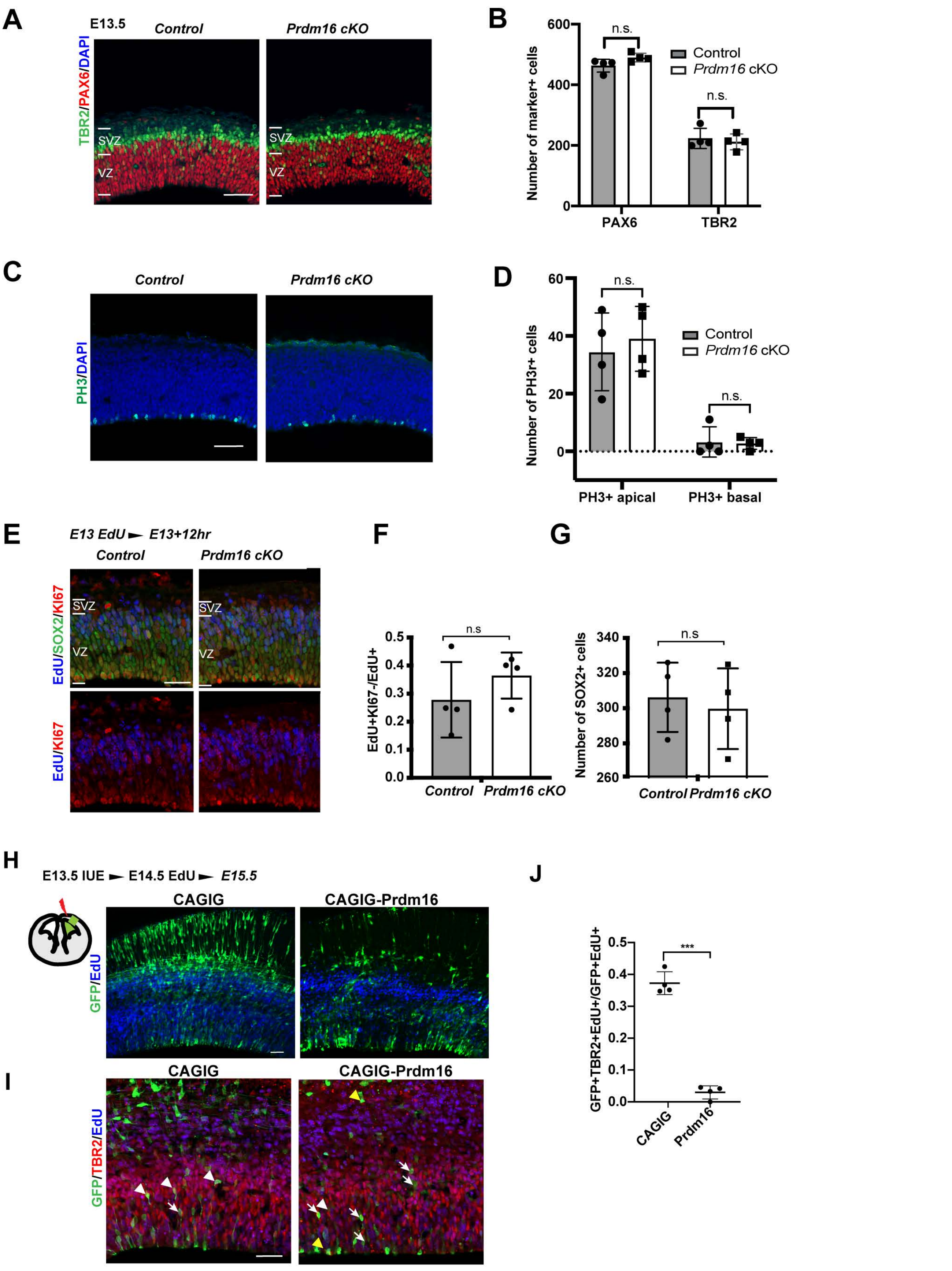

Development • Supplementary information

Figure S4: Depletion of PRDM16 does not affect RG proliferation at E13.5

(A) Images of E13.5 control and cKO brains, stained with Pax6 in red, Tbr2 in green and DAPI in blue. Scale bar: 50 µm. **(B)** Quantification of Pax6+ or Tbr2+ cells across the entire VZ and SVZ. **(C)** Images of E13.5 control and cKO brains, stained with the mitotic marker PH3. Scale bar: 50 µm. **(D)** Quantification of PH3+ cells across the entire VZ and SVZ. **(E)** Cell cycle exit analysis between E13 and E13.5. Scale bar: 50 µm. **(F)** Quantification of the fraction of Ki67-EdU+ relative to EdU+ cells, and (**G**) the fraction of SOX2 positive cells (n=4). (**H**) Images of E15.5 cortices from brains injected with CAGIG-PRDM16 or the CAGIG vector at E13.5. EdU admission was done at E14.5. Note: many GFP+ cells stuck at the ventricle surface and the IZ area in the mutant. (I) Higher magnification images from the same injections as in (H) stained with TBR2, GFP and EdU. White arrows and white arrowheads indicate GFP+EdU+ double positive cells and TBR2+GFP+EdU+ triple positive cells respectively. Yellow arrowheads highlight cells that showed abnormal processes. (**J**) Quantification of the fraction of TBR2+GFP+EdU+ cells among GFP+EdU+ cells (n=4). All data are shown as mean +/- SD; *$p<0.05$; **$p<0.01$; ***$p<0.001$; n.s., not significant.

He_Suppl.Fig. 5

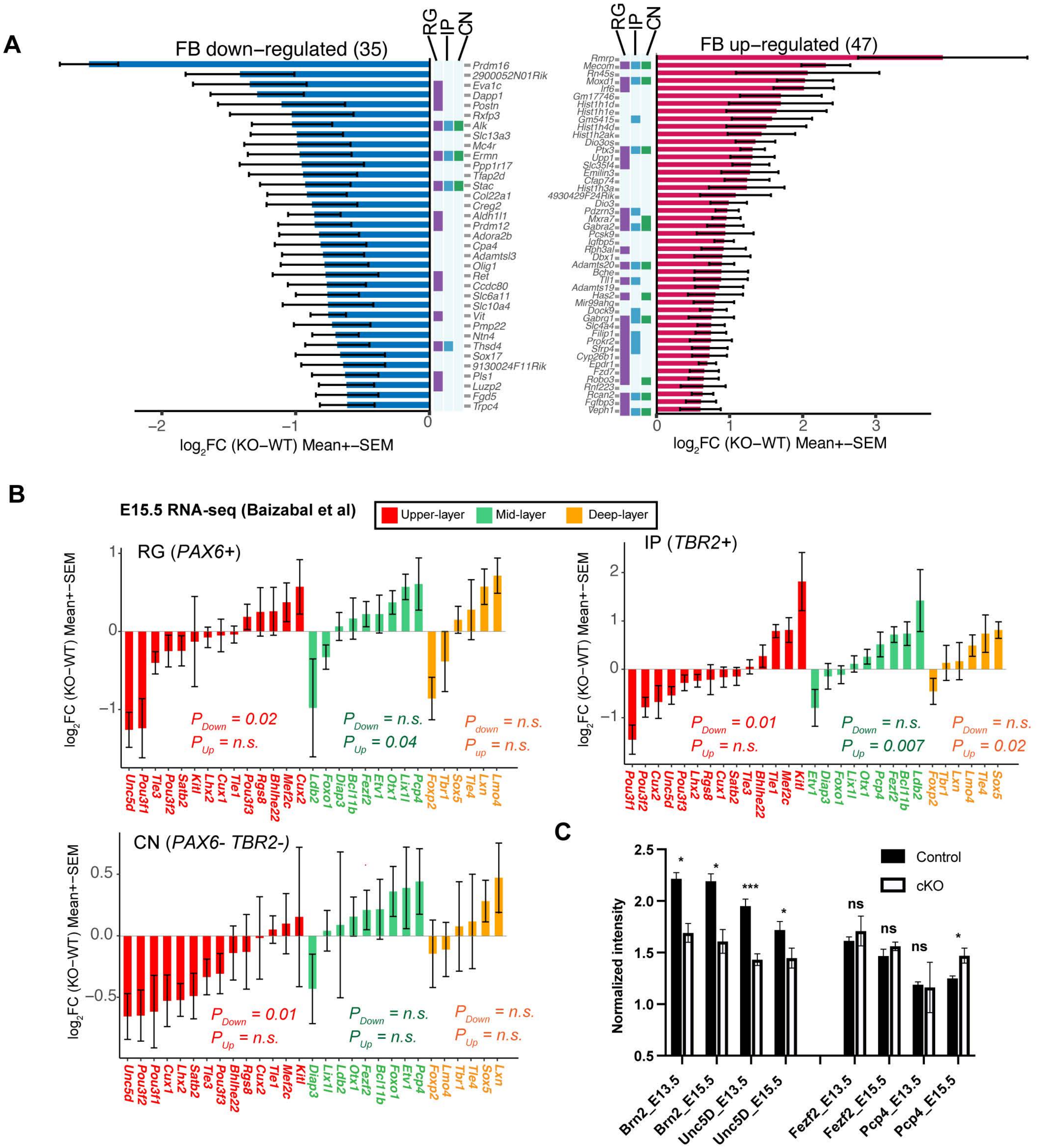

Development • Supplementary information

Figure S5: Top de-regulated genes in the mutant forebrains from RNA-seq analysis

(A) Bar graphs showing down-regulated genes in blue and up-regulated genes in red. Cutoff: Log_2FC (KO-WT) < 0.05. Purple, blue and green squares indicating genes that also changed expression in RG, IP and CN respectively from Baizabal *et al*, 2018. **(B)** Fold-changes of upper-layer, mid-layer, and deep-layer markers are shown for E15.5 RG, IP and CN (Baizabal et al). A gene set test was performed to test whether the layer markers are significantly changed as a set. The upper layer markers are significantly down-regulated in RG/IP. The mid-layer markers are significantly up-regulated in RG/IP. (**C**) Quantification of the signal intensity of in situ hybridization shown in Figure 4C. Signal from the VZ/SVZ area is normalized to a background area within the same image (n=3). All data are shown as mean +/- SD; *p<0.05; **p<0.01; ***p<0.001; ns: non-significant.

Development • Supplementary information

He_Suppl.Fig. 6

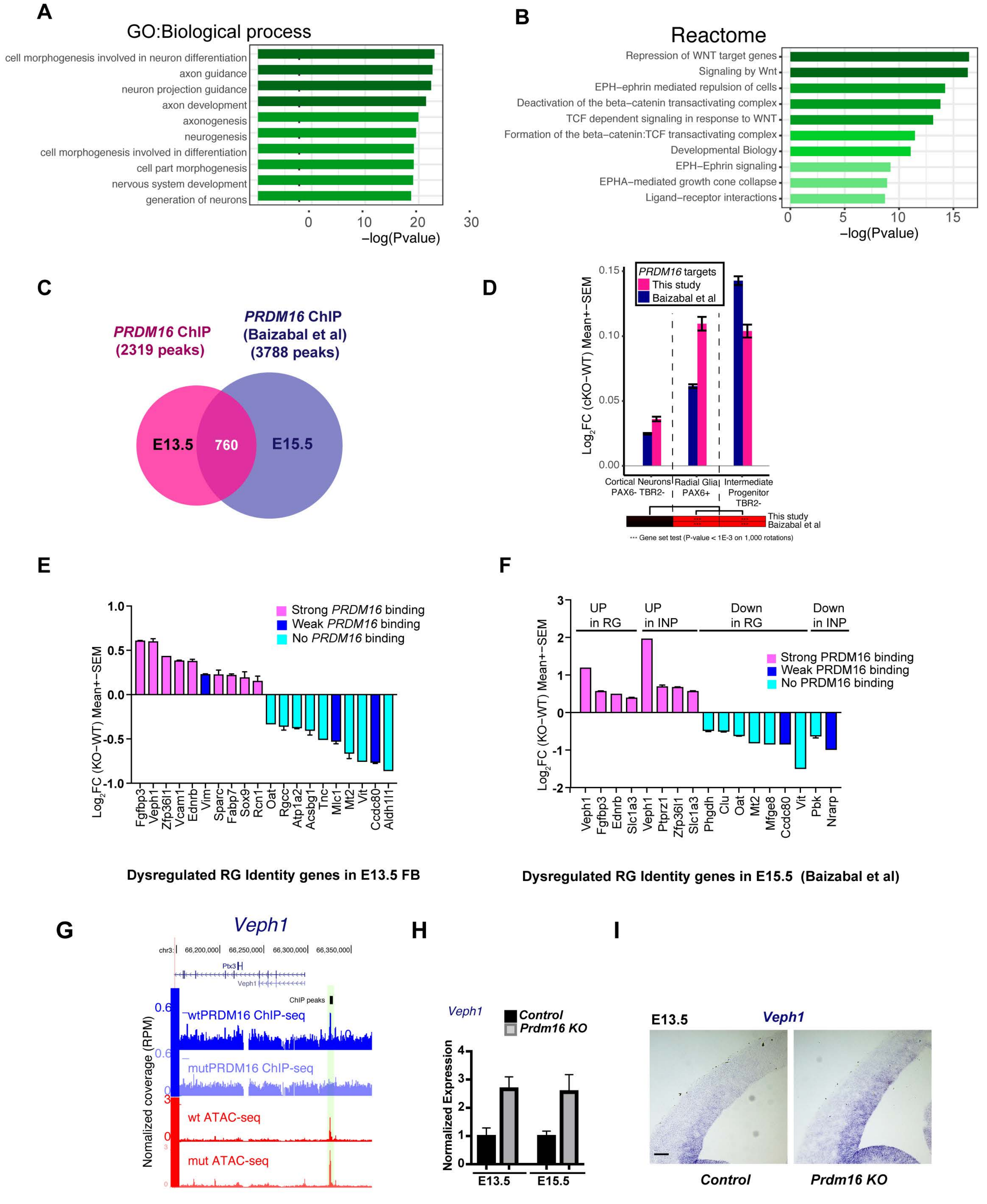

Figure S6: Target analyses from PRDM16 ChIP-seq and RNA-seq data.

PRDM16 peak functional enrichment are for **(A)** nervous system development in Gene Ontology (GO) "Biological Process", and **(B)** migration signaling pathways such as Eph signaling and semaphorin signaling and for RG function such as repression of WNT target genes in "Reactome". **(C)** Comparison of Prdm16 ChIP peaks in E13.5 whole brain (this study) and in E15.5 cortex (Baizabal et al). **(D)** Gene set testing shows that PRDM16 targets are significantly up-regulated in cKO vs control in RG and IP, but not in CN. **(E)** Plotting E13.5 FB RNA-seq data for the RG identity genes: those bound by PRDM16 show up-regulation in *Prdm16* mutant FB, while those weakly or not bound by PRDM16 were down-regulated. **(F)** Replotting E15.5 RNA-seq data (Baizabal et al. 2018): similarly, those bound by PRDM16 show up-regulation in *Prdm16* mutant RG or IP while those weakly (low peak score) or not bound by Prdm16 show down-regulation in *Prdm16* mutant cells. **(G)** Screenshot of the Veph1 gene locus, an example of bound and upregulated gene with increased chromatin accessibility**. (H-I)** RT-qPCR and *in situ* hybridization confirms de-repression of *Veph1* in E13.5 and E15.5 KO FB.

Development • Supplementary information

He_Suppl.Fig. 7

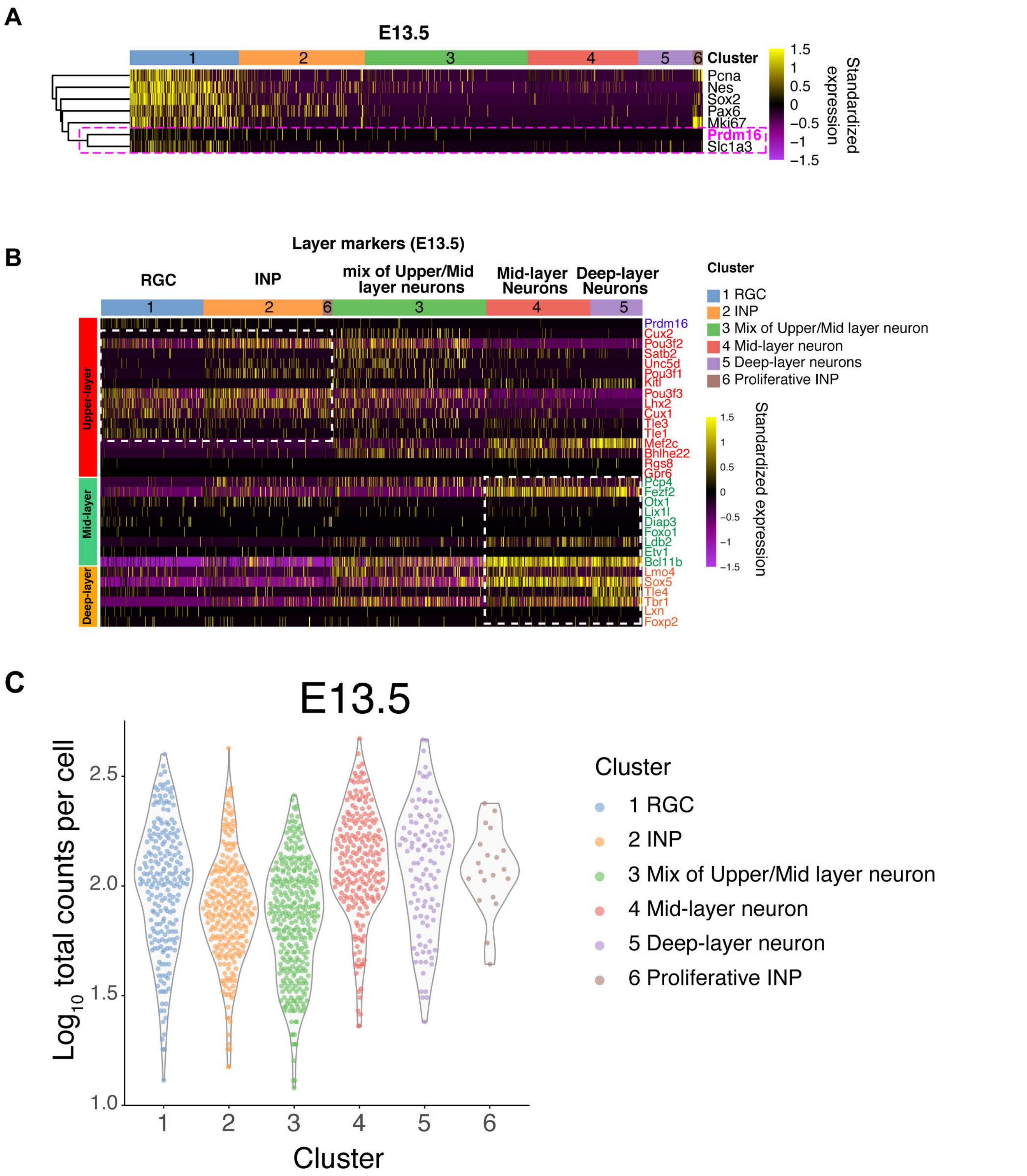

Figure S7: **(A)** *Prdm16* is mainly expressed in the RG clusters and is highly correlated with the RG marker *Slc1a3* at E13.5. **(B)** Single cell expression for upper-, mid- and deep-layer markers at E13.5. **(C)** Single cell expression for PRDM16 target genes at E13.5. Each dot in the violin represents the PRDM16 target expression (log10 total reads in each cell in each cluster).

Development • Supplementary information

He_Suppl.Fig. 8

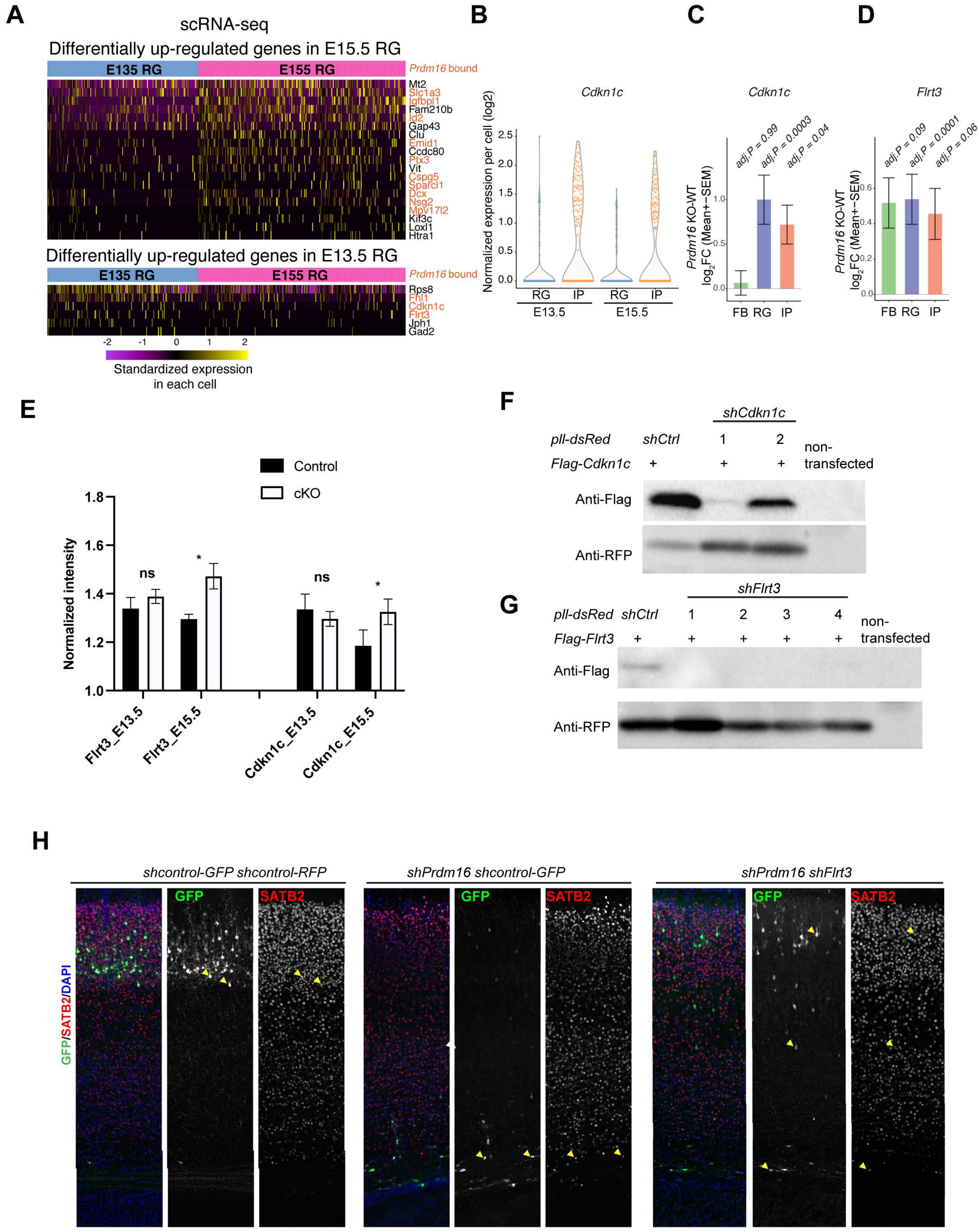

Development • Supplementary information

Figure S8. Analyses of temporally dynamic genes regulated by PRDM16

(A) Among differently expressed genes between E15.5 and E13.5 RG cells, 24 genes showed significant changes in *Prdm16* mutant RG (FDR <0.05). The heatmap of these 24 genes in the 13.5 (6 genes) and 15.5 RG (17 genes) scRNA-seq cells are shown. The genes that associate with PRDM16 ChIP peaks are highlighted in orange. (**B**) A violin plot shows normal expression of *Cdkn1c* in E13.5 and E15.5 RG and IPs. (**C-D**) Plotting gene expression change of the two PRDM16 targets, *Cdkn1c* and *Flrt3* from RNA-seq data. (**E**) Quantification of the signal intensity of in situ hybridization shown in Figure 7C. Signal from the VZ/SVZ area is normalized to a background area within the same image (n=3). All data are shown as mean +/- SD; *p<0.05; **p<0.01; ***p<0.001; ns non-significant. (**F**) Validation for the *Cdkn1c* shRNA constructs. *Cdkn1c* ShRNA1 efficiently reduced the co-expressed Flag-CDKN1C protein, and was selected for *in utero* electroporation experiments. (**G**) Validation for the *Flrt3* shRNA constructs. *Flrt3* shRNAs all efficiently reduced the co-expressed Flag-FLRT3 protein, and shRNA1 was used for *in utero* electroporation experiments. RFP is coded in the shRNA backbone and serves as an internal transfection control. (**H**) Staining for the upper-layer marker SATB2 on the brain slices with the three conditions of shRNA injections. Yellow arrowheads highlight some of the GFP and SATB2 double positive cells.

Development • Supplementary information

He_Suppl.Fig. 9

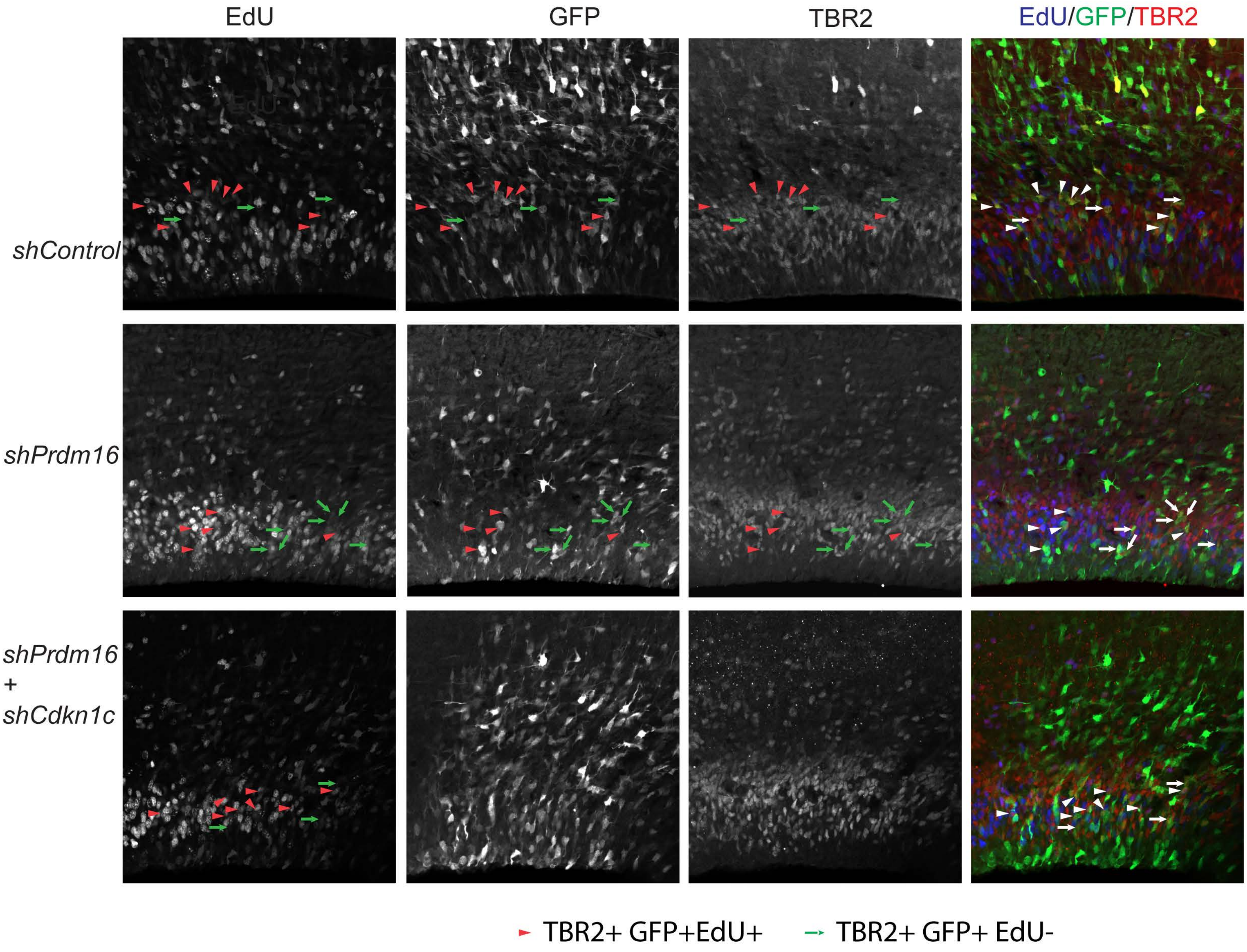

Figure S9. Single channel images of Figure 7G.

The arrowheads represent TBR2+EdU+GFP+ triple positive cells and the long green and white arrows indicates TBR2+GFP+ but EdU negative cells.

Development • Supplementary information

He_Suppl.Fig. 10

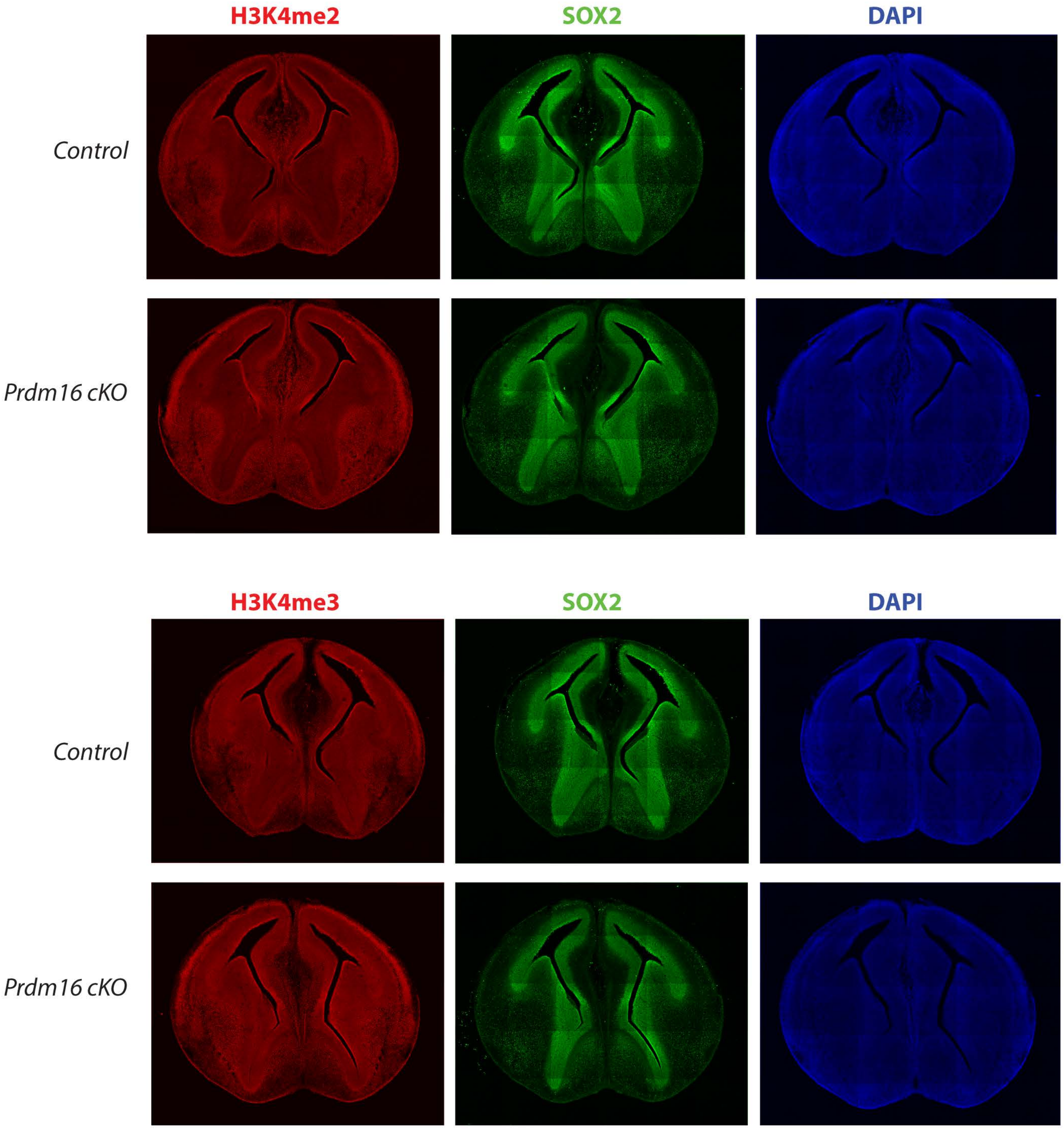

Figure S10: Loss of *Prdm16* in the cortex does not change H3K4 methylation level

Images from E14.5 control and *Prdm16* cKO brain slices, stained with SOX2, H3K4me2 and H3K4me3 antibodies and the DNA dye DAPI.

Development • Supplementary information
