# Supplementary material for: PRDM16 regulates a temporal transcriptional program to promote progression of cortical neural progenitors
Source: Development. 2021 Mar 17;148(6):dev194670. doi: 10.1242/dev.194670 (PMC7990860; doi:10.1242/dev.194670)
Supplement: Reviewer comments [file develop-148-194670_review_history.pdf]

PRDM16 regulates a temporal transcriptional program to promote progression of cortical neural progenitors

Li He, Jennifer Jones, Weiguo He, Bryan C Bjork, Jiayu Wen and Qi Dai
DOI: 10.1242/dev.194670

Editor: Paola Arlotta

Review timeline
Original submission: 3 July 2020
Editorial decision: 18 September 2020
First revision received: 4 December 2020
Editorial decision: 20 January 2021
Second revision received: 2 February 2021
Accepted: 11 February 2021

Original submission

First decision letter

MS ID#: DEVELOP/2020/194670

MS TITLE: Transcriptional program shifts temporally to promote progression of cortical neural progenitors

AUTHORS: Li He, Jennifer Jones, Weiguo He, Bryan C Bjork, Jiayu Wen, and Qi Dai

I have now received all the referees' reports on the above manuscript, and have reached a decision. The referees' comments are appended below, or you can access them online: please go to BenchPress and click on the 'Manuscripts with Decisions' queue in the Author Area.

As you will see, the referees express considerable interest in your work, but have some significant criticisms and recommend a substantial revision of your manuscript before we can consider publication. If you are able to revise the manuscript along the lines suggested, which may involve further experiments, I will be happy receive a revised version of the manuscript. Your revised paper will be re-reviewed by one or more of the original referees, and acceptance of your manuscript will depend on your addressing satisfactorily the reviewers' major concerns. Please also note that Development will normally permit only one round of major revision.

We are aware that you may be experiencing disruption to the normal running of your lab that make experimental revisions challenging. If it would be helpful, we encourage you to contact us to discuss your revision in greater detail. Please send us a point-by-point response indicating where you are able to address concerns raised (either experimentally or by changes to the text) and where you will not be able to do so within the normal timeframe of a revision. We will then provide further guidance. Please also note that we are happy to extend revision timeframes as necessary.

Please attend to all of the reviewers' comments and ensure that you clearly highlight all changes made in the revised manuscript. Please avoid using 'Tracked changes' in Word files as these are lost in PDF conversion. I should be grateful if you would also provide a point-by-point response detailing how you have dealt with the points raised by the reviewers in the 'Response to Reviewers' box. If you do not agree with any of their criticisms or suggestions please explain clearly why this is so.

© 2021. Published by The Company of Biologists under the terms of the Creative Commons Attribution License (https://creativecommons.org/licenses/by/4.0/).

Reviewer 1

Advance summary and potential significance to field

Recent work using next-generation sequencing techniques have highlighted that development from RG to their fully differentiated neuronal and glial progeny occurs trough a transcriptional continuous, allowing a surprisingly high degree of developmental flexibility. The histone methyltranferase PRDM16 and epigenetic modification of PRDM16-regulated enhancers seems crucial for the transcriptional priming of RG and the posterior differentiation and migration of RG neuronal progeny. In this context, the present work by He et al., use exhaustive similar and complementary approaches that largely confirm previous data showing the requirement of PRDM16 in choroid plexus development (Shimada et al., 2017), in cortical neurogenesis from RG, in the generation and proliferation of IP and in the production and migration of upper layers neurons (Baizabal et al 2018). As well as the principal role of PRDM16 in the neocortex as transcriptional repressor trough regulation of chromatin accessibility in distal enhancers and the requirement of PR domain. Nevertheless, there are some relevant new contributions: the identification of a set of genes in RG repressed by PRDM16 and necessary to balance direct and indirect neurogenesis and the transition from mid- to late-neurogenesis. In addition, this work demonstrate that PRDM16-dependent down regulation of three genes which expression declines in late neurogenesis; Fezf2, Cdkn1c and Flrt3, are necessary for the transition from mid- to late- neurogenesis, for maintain IP cell proliferation and for upper-layers neuron migration respectively.

Comments for the author

Additional comments
Page 2 lines 44-45: if I understood the message correctly, this sentence is contradictory “PRDM16 suppresses target gene expression maintaining chromatin accessibility of permissive enhancers”. Changing “maintaining” for “reducing or limiting” accessibility will make the sentence clearer.
Page 3 lines 86-87: “Radial glia at different neurogenesis stages may possess temporal gene expression programs in the RG…” Eliminate “in the RG” as it is repetitive.
Page 10 lines 329-330. The sentence “PRDM16 primarily acts as a repressor in RG trough maintaining accessibility of chromatin…” can be confusing. Changing “maintaining” for “reducing or limiting accessibility” will make the sentence clearer.
Page 29 line 1037: “mid—layer” change for “mid-layer”. Figure 7E: In the figure legends (page 30 lines 1051-), which represent solid black arrows and dotted red arrows in the wt RG graphic is no specified.

Reviewer 2

Advance summary and potential significance to field

In their manuscript titled "Transcriptional program shifts temporally to promote progression of cortical neural progenitors", Li He et al. generated a Prdm16 conditional knockout mouse. They found the role of Prdm16 on the temporal progression of radial glial cells, which was not addressed by the previous studies. The topic is interesting and important.

Comments for the author

The key finding was that 1) the excitatory cortical neurons labeled by thymidine analog at E14.5 ectopically expressed Ctip2, 2) the number of Tbr2-positive cells was reduced at E15.5 but not at E13.5, 3) the number of EdU-positive/Ki67-negative cells was increased, and that of EdU/Tbr2 double-positive cells was decreased in Prdm16 cKO at E15.5, 4) Cdkn1c and Flrt3 were identified as crucial target genes for IP proliferation and neuronal migration, respectively. Based on these data, they concluded that Prdm16 regulates the progression of radial glial cells producing layer V neurons, the generation and proliferation of IP cells producing upper-layer neurons, and the migration of upper-layer neurons. However, the reviewer could not agree on the part of their claims because of the reasons described in "major points".

© 2021. Published by The Company of Biologists under the terms of the Creative Commons Attribution License (https://creativecommons.org/licenses/by/4.0/).

Major points

1) The authors claimed that Prdm16 regulates IP cell proliferation based on the data of Fig. 3H, I. However since the number of Tbr2-positive cells was significantly reduced, the ratio of EdU/Tbr2 double-positive cells would decrease even if IP cells proliferate normally. They should check the alternative hypothesis that abnormal IP cell productions without affecting proliferation led the reduction of Tbr2-positive cells and Ki67-positive cells in the SVZ. They can address the alternative hypothesis by “(EdU+ Tbr2+)/Tbr2+” scoring instead of “(EdU+ Tbr2+)/EdU+”, and pH3 scoring at E15.5 in the SVZ.

2) On page 12, line 404, the authors describe "the shPrdm16 transfection decreased the number of TBR2+EdU+ cells", but they showed GFP+EdU+ in Fig. 7F-I. In this context, they should check Tbr2/EdU/GFP triple-positive cells to discuss IP cell proliferation if Cdkn1c regulates IP cell proliferation.

Minor points.

1) On page 9, line 280, "Expression of Pcp4 showed an increase in the mutant VZ and SVZ"; the Pcp4 signal looks decreased in Fig. 4C.

2) On page 28, line 988, 100 uM should be 100 um.

3) On page 28, line 994, the scale bar is not in Fig. 1H. Instead, scale bar information is missing in Fig. 1A.

4) In Fig. 4A; Pou3f3 and Bhlhe22 (upper-layer markers in Fig. 4B) is missing.

5) In Fig. 5A; The percentage looks not correct (40% -> 43%, 30% -> 33%, 20% -> 18%). Otherwise, the number of peak was not correct.

6) In page 30, line 1044, "Prdm16-FL-VP64 significantly induced expression of the Fezf2 reporter but not the empty pGL3-luc alone."; pGL-luc alone also induced Fezf2 reporter expression at some level.

7) "Prdm16" should be in the title.

<u>Reviewer 3</u>

Advance summary and potential significance to field

Paper summary:
In this paper, He et al. investigate the role of the transcriptional regulator PRDM16 in mouse cortical development. These experiments were carried out by analysing the cortical development phenotype of a PRDM16 cortex conditional knock-out as well in a complete knock-out animal. The authors find that there is reduced production of upper layer cortical neurons. Their investigations suggest that this phenotype is due to a defective mid to late neurogenesis transition due to a reduced production/proliferation/ specification of intermediate progenitor cells. They utilise RNAseq to show that loss of PRDM16 leads to a reduction of genes involved in the specification of upper layers at E13.5.

The authors demonstrate by CHIP-seq and ATACseq that PRDM16 acts as a transcriptional repressor in radial glia by binding to distal enhancers. They then show that PRDM16 binds to a Fezf2 regulatory enhancer element, and that it can repress Fezf2 expression in vitro. Cdkn1c and Flrt3 are then identified as genes bound by PRDM16, and which are derepressed in the mutant at E13.5 The authors show that knockdown of Cdkn1c and Flrt3 can partially rescue the IP cell phenotype and the migration phenotype repsectively.

© 2021. Published by The Company of Biologists under the terms of the Creative Commons Attribution License (https://creativecommons.org/licenses/by/4.0/).

General comments:
This paper is a well executed study. It should be noted the of the PRDM16 mutant phenotypes described in figure 1-3 have already been described by Baizabal et al 2018, so the initial findings are not a signifcant advance. I would argue however that it is encouraging to see experimental replication, and scientifically this should not be devalued as we need to encourage replication of findings in our field. The authors do add some novel findings, demonstrating a link between the PDRM16 bound genes Cdkn1c, Flrt3 and the IP cell phenotype and migration phenotype. This is an interesting further insight into the role of this gene in cortical development.

Comments for the author

I feel this paper contributes to the field and feel it is suitable for publication in development with revisions.

My suggestions to the authors to improve this paper are as follows:

Title:
-I feel this is overly vague and not specific enough to the findings of the paper. The title does not mention PRDM16, the main focus of the paper. It is also grammatically incorrect.

Fig 1/Fig S1:
-Could the authors show by Western blot that PRDM16 is absent in the conditional mutant and null cortex?
-Also, please show the greyscale image of PRDM16 in FigS1D, S1E as red is not a colour that is easy to see.

Fig2A:
-Please split the channels into greyscale panels to aid the visualisation of triple positive cells.
-There appears to be a significant Edu population in the null. Could the authors quantify their numbers? What is the fate of the Edu positive population? Do they make late born/upper layer cell types in the null?

Fig3C-D and Line 227: 'These result indicates that PRDM16 regulates RG division mode by promoting indirect neurogenesis.'

-Have the authors looked at the effects of overexpressing PRDM16 in vitro or in vivo (e.g. viral overexpression or electropration). Does this lead to increased numbers of TBR2?

Line 234: 'There was a significant increase of the percentage of Edu+Ki67- cells over all EdU+ cells in the VZ/SVZ region, indicating more cells exiting cell cycle in the mutant'. Could the authors use more specific language and explicitely state that the increase in Edu+Ki67- cells is in PRDM16 KO.

Line 260 and FigS4B: Could the authors comment more on the comparison of data RNA seq data between E13.5 and E15.5 from Baizabal et al 2018. Is comparing these two datasets really meaningful or fair comparison to make give the dynamic temporal changes in gene expression in the cortex?

Fig 4, line 254 onwards:
Could the authors be more explicit in the text as to their justification of choosing E13.5 for RNA seq and the following molecular studies, when most of the phenotype is observe later. While it may be obvious to experts, people with more general interest might not understand this.

Fig 4 C-D:
Please quantify the levels of mRNA expression observed in these sections. This is very important when making claims about expression levels.

Line 299 and Fig 5D:
See comment above about comparing E13.5 data with E15.5. Please comment further on whether this is a meaningful comparison.

© 2021. Published by The Company of Biologists under the terms of the Creative Commons Attribution License (https://creativecommons.org/licenses/by/4.0/).

Fig 5B:
It would be helpful to try to explain this figure more clearly. For example it is not immediately obvious what the top panels represent, or what mean RPM means?

Fig 6C, line 348:
-These data are from NSCs, but it would be more meaningful to demonstrate Fezf2 derepression in the mutant brains.
-The effect of derepression appears modest in figure 4B (RNAseq data). Could the authors comment on this?

Fig 7C:
-Quantifation of expression levels are necessary for the in situ-hybridisation sections

Fig 7F, line 404:
-No Tbr2 staining is shown here, can it be shown? It would be helpful to split these images into greyscale channels so that it is easier to see the phenotype being demonstrated.
-Does the double knockdown experiment rescue the upper layer neuron phenotype (Satb2) and mid layer (Ctip2) shown in fig 1? Could the authors quantify this?

Fig 7H
-What is the identity of the upper layer neurons where the migration phenotype is rescued. Are they Ctip2, or upper layer, or both?

-Have the authors tried a double knockdown of PRDM16 and Fezf2? Is any rescue phenotype observed?

-Have the authors tried to use their reporter assay from Fig 6 to demonstrate direct regulation of Cdkn1c and Flrt3 by PDRM16?

Minor comments:

-As a general comment please ensure that all figure legends appropriately explain the figure content and that all abreviations are annotated.
-Fig 3F 3I, x axes labels: cko cKo

First revision

Author response to reviewers' comments

Dear editors and reviewers,

We are grateful for the comments and the appreciation of the new findings beyond being able to replicate previous results. We are now submitting the revised manuscript, “PRDM16 regulates a temporal transcriptional program to promote progression of cortical neural progenitors”, to be considered for publication in Development.

We have included a substantial amount of new results in the current version of the manuscript to address the reviewers’ concerns.

Below is the point-to-point response.

Reviewer 1 Advance summary and potential significance to field

Recent work using next-generation sequencing techniques have highlighted that development from RG to their fully differentiated neuronal and glial progeny occurs trough a transcriptional continuous, allowing a surprisingly high degree of developmental flexibility. The histone

© 2021. Published by The Company of Biologists under the terms of the Creative Commons Attribution License (https://creativecommons.org/licenses/by/4.0/).

methyltranferase PRDM16 and epigenetic modification of PRDM16-regulated enhancers seems crucial for the transcriptional priming of RG and the posterior differentiation and migration of RG neuronal progeny. In this context, the present work by He et al., use exhaustive similar and complementary approaches that largely confirm previous data showing the requirement of PRDM16 in choroid plexus development (Shimada et al., 2017), in cortical neurogenesis from RG, in the generation and proliferation of IP and in the production and migration of upper layers neurons (Baizabal et al 2018). As well as the principal role of PRDM16 in the neocortex as transcriptional repressor trough regulation of chromatin accessibility in distal enhancers and the requirement of PR domain. Nevertheless, there are some relevant new contributions: the identification of a set of genes in RG repressed by PRDM16 and necessary to balance direct and indirect neurogenesis and the transition from mid- to late-neurogenesis. In addition, this work demonstrate that PRDM16-dependent down regulation of three genes which expression declines in late neurogenesis; Fezf2, Cdkn1c and Flrt3, are necessary for the transition from mid- to late- neurogenesis, for maintain IP cell proliferation and for upper-layers neuron migration respectively.

Reviewer 1 Comments for the author
Additional comments
Page 2 lines 44-45: if I understood the message correctly, this sentence is contradictory “PRDM16 suppresses target gene expression maintaining chromatin accessibility of permissive enhancers”. Changing “maintaining” for “reducing or limiting” accessibility will make the sentence clearer.
We appreciate the reviewer’s suggestion. We agree that “Limiting” is a better word to describe the function of PRDM16 in this context.

Page 3 lines 86-87: “Radial glia at different neurogenesis stages may possess temporal gene expression programs in the RG…” Eliminate “in the RG” as it is repetitive. We have changed the text accordingly.

Page 10 lines 329-330. The sentence “PRDM16 primarily acts as a repressor in RG trough maintaining accessibility of chromatin…” can be confusing. Changing “maintaining” for “reducing or limiting accessibility” will make the sentence clearer. We have changed the text accordingly.

Page 29 line 1037: “mid—layer” change for “mid-layer”. We have corrected the error.

Figure 7E: In the figure legends (page 30 lines 1051-), which represent solid black arrows and dotted red arrows in the wt RG graphic is no specified. Now the graph is reorganized in Figure 8. We have added this information in the revised manuscript.

Reviewer 2 Advance summary and potential significance to field
In their manuscript titled "Transcriptional program shifts temporally to promote progression of cortical neural progenitors", Li He et al. generated a Prdm16 conditional knockout mouse. They found the role of Prdm16 on the temporal progression of radial glial cells, which was not addressed by the previous studies. The topic is interesting and important.

Reviewer 2 Comments for the author
The key finding was that 1) the excitatory cortical neurons labeled by thymidine analog at E14.5 ectopically expressed Ctip2, 2) the number of Tbr2-positive cells was reduced at E15.5 but not at E13.5, 3) the number of EdU-positive/Ki67-negative cells was increased, and that of EdU/Tbr2 double-positive cells was decreased in Prdm16 cKO at E15.5, 4) Cdkn1c and Flrt3 were identified as crucial target genes for IP proliferation and neuronal migration, respectively. Based on these data, they concluded that Prdm16 regulates the progression of radial glial cells producing layer V neurons, the generation and proliferation of IP cells producing upper-layer neurons, and the migration of upper-layer neurons. However, the reviewer could not agree on the part of their claims because of the reasons described in "major points".

Major points

1) The authors claimed that Prdm16 regulates IP cell proliferation based on the data of Fig. 3H, I. However, since the number of Tbr2-positive cells was significantly reduced, the ratio of EdU/Tbr2 double-positive cells would decrease even if IP cells proliferate normally. They should check the

© 2021. Published by The Company of Biologists under the terms of the Creative Commons Attribution License (https://creativecommons.org/licenses/by/4.0/).

alternative hypothesis that abnormal IP cell productions without affecting proliferation led the reduction of Tbr2-positive cells and Ki67-positive cells in the SVZ. They can address the alternative hypothesis by “(EdU+ Tbr2+)/Tbr2+” scoring instead of “(EdU+ Tbr2+)/EdU+”, and pH3 scoring at E15.5 in the SVZ.
We appreciate the reviewer’s suggestion. We have done the new counting and also examined PH3 cell counts. The new results are in the revised manuscript (Fig. 3K). The fraction of TBR2EdU double positive cells versus TBR2 cells in the mutant is higher than that in the control. The PH3+ mitotic cells in the basal domain are also reduced in the mutant (Fig. 3L-M). These evidences indicate that loss of Prdm16 can lead to reduced IP cell proliferation.

2) On page 12, line 404, the authors describe "the shPrdm16 transfection decreased the number of TBR2+EdU+ cells", but they showed GFP+EdU+ in Fig. 7F-I. In this context, they should check Tbr2/EdU/GFP triple-positive cells to discuss IP cell proliferation if Cdkn1c regulates IP cell proliferation.
We appreciate the reviewer’s suggestion. We now have done the new counting and calculated the ratio of EdU+GFP+TBR2+ triple positive to GFP+TBR2+ double positive cells (Fig. 7H). shCdkn1c could rescue the reduction of proliferative IP cells induced by shPrdm16. Combined with the evidence that Prdm16 cKO animals show specific reduction in IP cell number, these results support that Cdkn1c is an important target gene regulated by PRDM16 in controlling IP cell proliferation.

Minor points.

1) On page 9, line 280, "Expression of Pcp4 showed an increase in the mutant VZ and SVZ"; the Pcp4 signal looks decreased in Fig. 4C. We have now quantified the signal intensity and confirmed that expression of Pcp4 is significantly increased in E15.5 mutant VZ/SVZ, while it is not significantly changed at E13.5. The new results are included in supplemental Fig S4C, and we have clarified the description in the text.

2) On page 28, line 988, 100 uM should be 100 um.
We appreciate the reviewer’s careful reading. We have changed it.

3) On page 28, line 994, the scale bar is not in Fig. 1H. Instead, scale bar information is missing in Fig. 1A.
We have changed it.

4) In Fig. 4A; Pou3f3 and Bhlhe22 (upper-layer markers in Fig. 4B) is missing.
We have added these genes in Fig. 4A.

5) In Fig. 5A; The percentage looks not correct (40% -> 43%, 30% -> 33%, 20% -> 18%). Otherwise, the number of peaks was not correct.
We have clarified the labeling with the precise percentage.

6) In page 30, line 1044, "Prdm16-FL-VP64 significantly induced expression of the Fezf2 reporter but not the empty pGL3-luc alone."; pGL-luc alone also induced Fezf2 reporter expression at some level. We appreciate the reviewer’s careful reading. We clarify the description by the new sentence, “Prdm16-FL-VP64 induced significantly higher expression of the Fezf2 reporter than the empty pGL3-luc alone.”

7) "Prdm16" should be in the title.
Now the title is “PRDM16 regulates a temporal transcriptional program to promote progression of cortical neural progenitors”.

Reviewer 3 Advance summary and potential significance to field
Paper summary:
In this paper, He et al. investigate the role of the transcriptional regulator PRDM16 in mouse cortical development. These experiments were carried out by analysing the cortical development phenotype of a PRDM16 cortex conditional knock-out as well in a complete knock-out animal. The authors find that there is reduced production of upper layer cortical neurons. Their investigations suggest that this phenotype is due to a defective mid to late neurogenesis transition due to a

© 2021. Published by The Company of Biologists under the terms of the Creative Commons Attribution License (https://creativecommons.org/licenses/by/4.0/).

reduced production/proliferation/ specification of intermediate progenitor cells. They utilise RNAseq to show that loss of PRDM16 leads to a reduction of genes involved in the specification of upper layers at E13.5.The authors demonstrate by CHIP-seq and ATACseq that PRDM16 acts as a transcriptional repressor in radial glia by binding to distal enhancers. They then show that PRDM16 binds to a Fezf2 regulatory enhancer element, and that it can repress Fezf2 expression in vitro. Cdkn1c and Flrt3 are then identified as genes bound by PRDM16, and which are derepressed in the mutant at E13.5 The authors show that knockdown of Cdkn1c and Flrt3 can partially rescue the IP cell phenotype and the migration phenotype repsectively.

General comments:
This paper is a well executed study. It should be noted the of the PRDM16 mutant phenotypes described in figure 1-3 have already been described by Baizabal et al 2018, so the initial findings are not a signifcant advance. I would argue however that it is encouraging to see experimental replication, and scientifically this should not be devalued as we need to encourage replication of findings in our field. The authors do add some novel findings, demonstrating a link between the PDRM16 bound genes Cdkn1c, Flrt3 and the IP cell phenotype and migration phenotype. This is an interesting further insight into the role of this gene in cortical development.

Reviewer 3 Comments for the author
I feel this paper contributes to the field and feel it is suitable for publication in development with revisions.

My suggestions to the authors to improve this paper are as follows:

Title:
-I feel this is overly vague and not specific enough to the findings of the paper. The title does not mention PRDM16, the main focus of the paper. It is also grammatically incorrect.
Yes, we have revised the title to, “PRDM16 regulates a temporal transcriptional program to promote progression of cortical neural progenitors”.

Fig 1/Fig S1:
-Could the authors show by Western blot that PRDM16 is absent in the conditional mutant and null cortex?
We appreciate the reviewer’s suggestion. Prior to the previous submission to Development, we have tested all the antibodies we have obtained either from our collaborator Bryan Bjork or from DHSB work, but none of them worked on Western-Blot. However, we have validated the knockout using genotyping (Strassman, et al 2017 and this study) and immunostaining (this study) (Supplementary Fig 1D-E, 1H-I) and RT-qPCR (Fig. 1C). All of these methods showed the loss of Prdm16 gene product in the entire animal in the cGT null condition or specifically in the forebrain in the Emx1-Cre conditional KO.

-Also, please show the greyscale image of PRDM16 in FigS1D, S1E as red is not a colour that is easy to see.
Yes, we have changed the PRDM16 channel into grey.

Fig2A:
-Please split the channels into greyscale panels to aid the visualisation of triple positive cells.
We have added additional image panels to this figure (Fig. 2A). Some of the CTIP2 and BrdU double positive cells are highlighted with arrowheads. There are more CTIP2+BrdU+ double positive cells in the mutant cortex than in the control.

-There appears to be a significant Edu population in the null. Could the authors quantify their numbers? What is the fate of the Edu positive population? Do they make late born/upper layer cell types in the null?
These EdU+ cells are later born neurons, labeled with a later born marker CUX1. The images were shown in Fig S2C. We now have clarified the description in the revised manuscript.

Fig3C-D and Line 227: 'These result indicates that PRDM16 regulates RG division mode by promoting indirect neurogenesis.'

© 2021. Published by The Company of Biologists under the terms of the Creative Commons Attribution License (https://creativecommons.org/licenses/by/4.0/).

-Have the authors looked at the effects of overexpressing PRDM16 in vitro or in vivo (e.g. viral overexpression or electropration). Does this lead to increased numbers of TBR2?
Upon the reviewer's suggestion, we have looked the overexpression effects of PRDM16 on TBR2 cell number, by injecting a PRDM16 overexpression construct in utero. Overexpression of Prdm16 caused severe migration defect, consistent with the finding in a previous study (Inoue M et al., 2017, Development), and dramatically reduced the number of TBR2 positive cells. This means that the right expression level of PRDM16 is important for both neuronal migration and IP cell production. This result suggests that PRDM16 is in a complex regulatory network, and thus too much or too little PRDM16 protein will lead to the disruption of the network required for normal behavior of RG. The new data is presented in Fig. S3H-J.

Line 234: 'There was a significant increase of the percentage of Edu+Ki67- cells over all EdU+ cells in the VZ/SVZ region, indicating more cells exiting cell cycle in the mutant'. Could the authors use more specific language and explicitely state that the increase in Edu+Ki67- cells is in PRDM16 KO.
We apologize for the confusion. We have clarified this in the revised manuscript, by changing the sentence to " Compared with the control, the Prdm16 cKO cortex displayed a significant increase…"

Line 260 and FigS4B: Could the authors comment more on the comparison of data RNA seq data between E13.5 and E15.5 from Baizabal et al 2018. Is comparing these two datasets really meaningful or fair comparison to make give the dynamic temporal changes in gene expression in the cortex?
There is for sure limitation of the comparison, as our E13.5 RNA-seq data was from whole forebrain while the E15.5 data was from sorted three cell types. For genes that are changed in only one of the two datasets, we cannot make any conclusion because the difference can come from stage difference, technical difference etc. So we only commented on genes that are consistently deregulated in both datasets (Supplemental S4A). For the analysis in S4B, we would like to use it as an additional control to show that some of layer-specific genes are also de-regulated in the published datasets. There is no direct comparison between our E13.5 data and the E15.5 data.

Fig 4, line 254 onwards:
Could the authors be more explicit in the text as to their justification of choosing E13.5 for RNA seq and the following molecular studies, when most of the phenotype is observe later. While it may be obvious to experts, people with more general interest might not understand this.
E13.5 is the peak time when radial glia produce mid-layer neurons. Because there is higher number of mid-layer neurons in Prdm16 mutant cortex and PRDM16 is expressed in radial glia but not mid-layer neurons, we expect to see a direct effect on gene expression in the mutant. If we had profiled RNA-seq from the whole cortex in a later stage, we might have missed direct effect and instead more likely obtained de-regulated genes that are consequences of cell fate change. We now add this description to the text "Because the cell fate change in Prdm16 mutant occurs at the transition of mid-layer neuron production and upper-layer neuron production (Fig. 2), we hypothesized that a direct regulatory role of PRDM16 on RG gene expression is critical at E13.5. To this end, …"

Fig 4 C-D:
Please quantify the levels of mRNA expression observed in these sections. This is very important when making claims about expression levels.
We appreciate the reviewer's suggestion. We have done the quantification using imageJ and present the result in the revised manuscript (Supplemental Fig. S4C).

Line 299 and Fig 5D:
See comment above about comparing E13.5 data with E15.5. Please comment further on whether this is a meaningful comparison.
Similar to the explanation above, we do not compare E13.5 with E15.5. We use the published E15.5 data as a confirmative dataset to show in both stages, genes that are associated with more open chromatin become de-repressed.
Fig 5B:
It would be helpful to try to explain this figure more clearly. For example it is not immediately obvious what the top panels represent, or what mean RPM means?
We have now added the description "To examine accessibility of PRDM16-bound regions, we plotted mean reads per million (RPM) of PRDM16 ChIP peaks in a metaplot and ranked the peaks in a

© 2021. Published by The Company of Biologists under the terms of the Creative Commons Attribution License (https://creativecommons.org/licenses/by/4.0/).

heatmap, and then compared the ATAC-seq signal intensity in wt and mutant for each peak (Fig. 5B).” to the text.

Fig 6C, line 348:
-These data are from NSCs, but it would be more meaningful to demonstrate Fezf2 derepression in the mutant brains.
We have examined Fezf2 expression in the mutant brains by in situ hybridization (Fig4C) and qPCR from the whole cortex (Fig. 1C). The whole cortex contains all three major cell types, RG, INP and neurons, so RT-qPCR from the whole tissue could only reflect cell fate change. We also tried to detect the mRNA change using in situ hybridization, but found no obvious change in Fezf2 signal at E13.5 and E15.5 in mutant brain (Fig 4C-D and the new Supplemental S4C). We reasoned that this could be due to the limitation of the method in resolution. For example, if expression of Fezf2 changes with a modest increase in a small population of mutant cells in the cortex, in situ experiment may not be able to detect the change. To overcome this issue, we performed RT-qPCR on primary NSC culture isolated from control and mutant cortices (Fig 6C), and observed that there is a significant increase of Fezf2 expression.

-The effect of derepression appears modest in figure 4B (RNAseq data). Could the authors comment on this?

We think the modest effect could be due to two reasons. First, the limitation of the samples. The RNA samples for RNA-seq were from the whole forebrain tissue which contains the RG, IP and neuronal cell types. At E13.5, the forebrain already contains deep-layer and mid-layer neurons. The mid-layer marker genes, including Fezf2, are also expressed in the deep layer. As Prdm16 deletion does not affect the population of deep-layer neurons, the presence of mid-layer gene products in deep-layer neurons will partly mask gene expression change that occurs in RG and mid-layer neurons. Second, the expression change of mid-layer neuron genes in the mutant is not dramatic. This is consistent with the phenotypic analysis (Fig. 2) that there is a prolonged production of mid-layer neurons, while the overall extent is mild (compare BrdU Ctip2 double positive cells with Ctip2 single positive cells). Thus, we do not expect that expression of Fezf2 has a dramatic increase.

Fig 7C:
-Quantifation of expression levels are necessary for the in situ-hybridisation sections
We have done the quantification and present the result in the revised manuscript (Supplemental Fig. S7E).

Fig 7F, line 404:
-No Tbr2 staining is shown here, can it be shown? It would be helpful to split these images into greyscale channels so that it is easier to see the phenotype being demonstrated.
Yes, we now have added a TBR2 panel and calculated the fraction of GFP+TBR2+EdU+ triple positive cells versus GFP+TBR2+ double positive cells to assess the effect on IP cell proliferation. Because the greyscale channels cannot show double and triple positive cells, we think that these images cannot help to see the phenotypes. Due to the space limitation in figure 7, we decide not to include these grayscale channels. Instead, we indicate some of these cells using arrowheads and arrows in the images. However, if the reviewer wants to see the images, we can provide the images in a separate document.
-Does the double knockdown experiment rescue the upper layer neuron phenotype (Satb2) and mid layer (Ctip2) shown in fig 1? Could the authors quantify this?
We do not expect double knockdown of Cdkn1c and Prdm16 could rescue the cell fate change phenotype. Based on our phenotypic analysis and genomic data, we think that PRDM16 regulates several sets of target genes that are responsible for its function in mid- to upper- layer neuron fate transition. Among these, Cdkn1c is responsible for regulating IP cell proliferation.

Fig 7H
-What is the identity of the upper layer neurons where the migration phenotype is rescued. Are they Ctip2, or upper layer, or both?
We have done immunostainings and confirmed that the GFP+ cells are upper layer neurons. We now include the result in the revised manuscript (Supplementary Fig. S7H).

© 2021. Published by The Company of Biologists under the terms of the Creative Commons Attribution License (https://creativecommons.org/licenses/by/4.0/).

-Have the authors tried a double knockdown of PRDM16 and Fezf2? Is any rescue phenotype observed?
Yes, we have tried Prdm16 single knockdown (KD) as well as Prdm16 and Fezf2 double KD at E13, E13.5 and E14. However, we could not detect a clear effect on CTIP2 cells by shPRDM16 alone or shFezf2+shPrdm16. At E13 and E13.5, the transfected cells with the sh-control, shPrdm16 or shPrdm16+shFezf2 were all labeled with CTIP2. At E14, none of the transfected cells expresses CTIP2. There could be two explanations. First, it may be very difficult to catch the right transition point to shift cell fates by injecting knockdown constructs. Second, compared with complete removal of Prdm16 in the Prdm16 cKO animal, reducing Prdm16 level by shPrdm16 is insufficient to delay neuronal cell fate transition.

-Have the authors tried to use their reporter assay from Fig 6 to demonstrate direct regulation of Cdkn1c and Flrt3 by PDRM16?
Corresponding to the suggestion, we have cloned and tested a series of reporters spanning the two gene regions. We identified two enhancer regions in Cdkn1c and one in Flrt3 that are responsive to PRDM16-VP64 fusion protein in luciferase reporter assays (new Fig. 7D-F).
Minor comments:

-As a general comment please ensure that all figure legends appropriately explain the figure content and that all abreviations are annotated.
We appreciate the reviewer's careful reading. We have tried to clarify all labeling in the revised manuscript.

-Fig 3F 3I, x axes labels: cko cKo
We have corrected the labeling in the revised manuscript.

Second decision letter

MS ID#: DEVELOP/2020/194670

MS TITLE: PRDM16 regulates a temporal transcriptional program to promote progression of cortical neural progenitors

AUTHORS: Li He, Jennifer Jones, Weiguo He, Bryan C Bjork, Jiayu Wen, and Qi Dai

I am sorry for taking a long time to receive all the reviews back. I have now received all the referees reports on the above manuscript, and have reached a decision. The referees' comments are appended below, or you can access them online: please go to BenchPress and click on the 'Manuscripts with Decisions' queue in the Author Area.

The overall evaluation is positive and we would like to publish a revised manuscript in Development, provided that you can address the comment of Reviewer 2 about the quality of some of the images. Please attend to all of the reviewers' comments in your revised manuscript and detail them in your point-by-point response. If you do not agree with any of their criticisms or suggestions explain clearly why this is so.

We are aware that you may currently be unable to access the lab to undertake experimental revisions. If it would be helpful, we encourage you to contact us to discuss your revision in greater detail. Please send us a point-by-point response indicating where you are able to address concerns raised (either experimentally or by changes to the text) and where you will not be able to do so within the normal timeframe of a revision. We will then provide further guidance. Please also note that we are happy to extend revision timeframes as necessary.

Reviewer 1

Advance summary and potential significance to field

© 2021. Published by The Company of Biologists under the terms of the Creative Commons Attribution License (https://creativecommons.org/licenses/by/4.0/).

In this paper, He et al., corroborate previous data showing the requirement of PRDM16 in choroid plexus development (Shimada et al., 2017) and in cortical neurogenesis from RG, in particular the generation and proliferation of IP and the production and migration of upper layers neurons (Baizabal et al 2018) and extend the previous work adding relevant new contributions. With respect the previous version of the manuscript, the authors have better sustained their statements with new quantifications and complementary experiments; added relevant new data as the precise dose-dependency of PRDM16 for correct IP and the identification of new enhancer regions in Cdknic and Flrt3 responsive for PRDM16; and have answer most of the refereeÂ's questions, which together significantly improves the impact and relevance of their work. In addition, the manuscript is now clearer and much easier to read.

Comments for the author

Minor comments:
Page 5 line 136: "regulation on progenitor proliferation…" should be "regulation of progenitor proliferation…"
Page 6 line 184 and page 539: eliminate capital letters in "Heterotopia"
Page 7 line 225: check bibliography format.
Page 8 line 251: "Fig. S3E-G" should be "Fig. S3E"
Page 10 line 313: "found around 30% (798)… (Fig. S5C)". In Fig. S5C the number of genes is 760. Please check which number is the correct one.
Page 12 lines 384-385: "120 and 248 genes were identified that show higher expression at…" "120 and 248 genes were identified that show higher expression at…" change by "We identified 120 and 248 genes with higher expression at…" or similar.
Page 16 line 538: "by which Prdm16 loss of function in the formation of Heterotopia, a…" The sentence is incomplete, may be the authors would say that Prdm16 loss of function is involved, related or subjacent to the formation of heterotopia.
Page 23 lines 764-765: remove capital letters from "OR"

<u>Reviewer 2</u>

Advance summary and potential significance to field

The topic is interesting and important.

Comments for the author

In this revised manuscript, the authors perfectly addressed the concern of the reviewer. However, the reviewer asks to improve the figure images.

It is still hard to convince Fig. 3I and 7H from the images in Fig. 3E and 7G, respectively. The reviewer asks the authors to show large greyscale panels in a supplemental figure, as Reviewer 3 suggested. Multiple greyscale images with an arrow would be nice to distinguish single-, double-, and triple-positive cells.

<u>Reviewer 3</u>

Advance summary and potential significance to field

In this paper, He et al. investigate the role of the transcriptional regulator PRDM16 in mouse cortical development. These experiments were carried out by analysing the cortical development phenotype of a PRDM16 cortex conditional knock-out as well in a complete knock-out animal. The authors find that there is reduced production of upper layer cortical neurons. Their investigations suggest that this phenotype is due to a defective mid to late neurogenesis transition due to a reduced production/proliferation/ specification of intermediate progenitor cells. They utilise RNAseq to show that loss of PRDM16 leads to a reduction of genes involved in the specification of upper layers at E13.5.The authors demonstrate by CHIP-seq and ATACseq that PRDM16 acts as a

© 2021. Published by The Company of Biologists under the terms of the Creative Commons Attribution License (https://creativecommons.org/licenses/by/4.0/).

transcriptional repressor in radial glia by binding to distal enhancers. They then show that PRDM16 binds to a Fezf2 regulatory enhancer element, and that it can repress Fezf2 expression in vitro. Cdkn1c and Flrt3 are then identified as genes bound by PRDM16, and which are derepressed in the mutant at E13.5 The authors show that knockdown of Cdkn1c and Flrt3 can partially rescue the IP cell phenotype and the migration phenotype repsectively.

Comments for the author

The authors have added to this substantial body of work with a number of important experiments which strengthen the evidence and this body of work. They have satisfactorily addressed the majority of my comments.

Second revision

Author response to reviewers' comments

Dear editors and reviewers,

We are grateful for the reviewers' additional comments. We are now submitting a further revised manuscript, "PRDM16 regulates a temporal transcriptional program to promote progression of cortical neural progenitors", to be considered for publication in Development.

We have added two more supplementary figures to show single channel images upon reviewer 2's suggestion in the current version of the manuscript and corrected the mistakes pointed out by reviewer 1.

Below is the point-to-point response.

Reviewer 1 Advance summary and potential significance to field
In this paper, He et al., corroborate previous data showing the requirement of PRDM16 in choroid plexus development (Shimada et al., 2017) and in cortical neurogenesis from RG, in particular the generation and proliferation of IP and the production and migration of upper layers neurons (Baizabal et al 2018) and extend the previous work adding relevant new contributions. With respect the previous version of the manuscript, the authors have better sustained their statements with new quantifications and complementary experiments; added relevant new data as the precise dose-dependency of PRDM16 for correct IP and the identification of new enhancer regions in Cdknic and Flrt3 responsive for PRDM16; and have answer most of the referee's questions, which together significantly improves the impact and relevance of their work. In addition, the manuscript is now clearer and much easier to read.

We are grateful that the reviewer thinks our manuscript has been improved.

Reviewer 1 Comments for the author
Minor comments:
Page 5 line 136: "regulation on progenitor proliferation…" should be "regulation of progenitor proliferation…"
Page 6 line 184 and page 539: eliminate capital letters in "Heterotopia"
Page 7 line 225: check bibliography format.
Page 8 line 251: "Fig. S3E-G" should be "Fig. S3E"
Page 10 line 313: "found around 30% (798)… (Fig. S5C)". In Fig. S5C the number of genes is 760. Please check which number is the correct one.
Page 12 lines 384-385: "120 and 248 genes were identified that show higher expression at…" "120 and 248 genes were identified that show higher expression at…" change by "We identified 120 and 248 genes with higher expression at…" or similar.
Page 16 line 538: "by which Prdm16 loss of function in the formation of Heterotopia, a…" The sentence is incomplete, may be the authors would say that Prdm16 loss of function is involved, related or subjacent to the formation of heterotopia.

© 2021. Published by The Company of Biologists under the terms of the Creative Commons Attribution License (https://creativecommons.org/licenses/by/4.0/).

Page 23 lines 764-765: remove capital letters from “OR”

We appreciate the reviewer’s careful reading. We have corrected all of these mistakes in the revised version.

Reviewer 2 Advance summary and potential significance to field
The topic is interesting and important.

Reviewer 2 Comments for the author
In this revised manuscript, the authors perfectly addressed the concern of the reviewer. However, the reviewer asks to improve the figure images.

It is still hard to convince Fig. 3I and 7H from the images in Fig. 3E and 7G, respectively. The reviewer asks the authors to show large greyscale panels in a supplemental figure, as Reviewer 3 suggested. Multiple greyscale images with an arrow would be nice to distinguish single-, double-, and triple-positive cells.

As the Fig. 3I represents the image in Fig. 3H, we assume that the reviewer is asking us to show single channel images of Fig.3H and and Fig. 7G. We have now included two additional supplementary figures, Figure S3 and Figure S9, to show single channel images. We also highlighted the relevant cell types according to the reviewer’s suggestion.

Reviewer 3 Advance summary and potential significance to field
In this paper, He et al. investigate the role of the transcriptional regulator PRDM16 in mouse cortical development. These experiments were carried out by analysing the cortical development phenotype of a PRDM16 cortex conditional knock-out as well in a complete knock-out animal. The authors find that there is reduced production of upper layer cortical neurons. Their investigations suggest that this phenotype is due to a defective mid to late neurogenesis transition due to a reduced production/proliferation/ specification of intermediate progenitor cells. They utilise RNAseq to show that loss of PRDM16 leads to a reduction of genes involved in the specification of upper layers at E13.5.The authors demonstrate by CHIP-seq and ATACseq that PRDM16 acts as a transcriptional repressor in radial glia by binding to distal enhancers. They then show that PRDM16 binds to a Fezf2 regulatory enhancer element, and that it can repress Fezf2 expression in vitro. Cdkn1c and Flrt3 are then identified as genes bound by PRDM16, and which are derepressed in the mutant at E13.5 The authors show that knockdown of Cdkn1c and Flrt3 can partially rescue the IP cell phenotype and the migration phenotype repsectively.

Reviewer 3 Comments for the author
The authors have added to this substantial body of work with a number of important experiments which strengthen the evidence and this body of work. They have satisfactorily addressed the majority of my comments.

We are grateful that the reviewer thinks our manuscript has been improved.

Third decision letter

MS ID#: DEVELOP/2020/194670

MS TITLE: PRDM16 regulates a temporal transcriptional program to promote progression of cortical neural progenitors

AUTHORS: Li He, Jennifer Jones, Weiguo He, Bryan C Bjork, Jiayu Wen, and Qi Dai
ARTICLE TYPE: Research Article

I am happy to tell you that your manuscript has been accepted for publication in Development, pending our standard ethics checks.

© 2021. Published by The Company of Biologists under the terms of the Creative Commons Attribution License (https://creativecommons.org/licenses/by/4.0/).
